# *In Vitro* Antibacterial and Antioxidant Activities, Pharmacokinetics, and *In Silico* Molecular Docking Study of Phytochemicals from the Roots of *Ziziphus spina-christi*

**DOI:** 10.1155/2024/7551813

**Published:** 2024-08-09

**Authors:** Hadush Gebrehiwot, Urgessa Ensermu, Aman Dekebo, Milkyas Endale, Tariku Nefo Duke

**Affiliations:** ^1^ Department of Applied Chemistry Adama Science and Technology University, P.O. Box 1888, Adama, Ethiopia; ^2^ Department of Applied Biology Adama Science and Technology University, P.O. Box 1888, Adama, Ethiopia; ^3^ Institute of Pharmaceutical Sciences Adama Science and Technology University, P.O. Box 1888, Adama, Ethiopia; ^4^ Traditional and Modern Medicine Research and Development Directorate Armauer Hansen Research Institute, P.O. Box 1242, Addis Ababa, Ethiopia; ^5^ Department of Materials Science and Engineering National Taiwan University of Science and Technology, No. 43, Keelung Rd., Sec. 4, Taipei 10607, Taiwan

## Abstract

*Ziziphus spina-christi* (Rhamnaceae family) is a medicinal plant traditionally used to treat dandruff, wounds, hair loss, diarrhea, mastitis, abdominal pain, and gastrointestinal complications. To support this, the present work aims to study the *in vitro* antibacterial and antioxidant activities of compound isolates from the roots of *Ziziphus spina-christi* along with their *in silico* computational analyses. Compounds were isolated on silica gel column chromatography and an agar disc diffusion and DPPH radical scavenging assays were employed to study the antibacterial and antioxidant activities, respectively. The ADME and toxicity properties of the compounds were evaluated using SwissADME and ProTox-II online Web tools, respectively. Conversely, the *in silico* molecular docking studies were attained via a Biovia Discovery Studio Visualizer 2021 in combination with the AutoDock Vina software. The silica gel chromatographic separation of the combined CH_2_Cl_2_ : CH_3_OH (1 : 1) and CH_3_OH root extracts afforded trimethyl trilinolein (**1**), stearic acid (**2**), 13-hydroxyoctadeca-9, 11-dienoic acid (**3**), *β*-sitosteryl-3*β*-glucopyranoside-6′-*O*-palmitate (**4**), and stigmasterol (**5**). Notably, the *in vitro* antibacterial study revealed the extract and *β*-sitosteryl-3*β*-glucopyranoside-6′-*O*-palmitate (**4**) with the highest inhibitory activities (15.25 ± 0.35 and 14.25 ± 0.35 mm, respectively) against *E. coli* compared to ciprofloxacin (21.00 ± 0.35 mm) at 2 mg/mL. The CH_2_Cl_2_ : CH_3_OH (1 : 1) extract (IC_50_ : 1.51 *µ*g/mL) and *β*-sitosteryl-3*β*-glucopyranoside-6′-*O*-palmitate (**4**) (IC_50_ : 5.41 *µ*g/mL) also exhibited auspicious DPPH scavenging activities, followed by stigmasterol (**5**) (IC_50_ : 6.88 *µ*g/mL) compared to the ascorbic acid standard (IC_50_ : 0.46 *µ*g/mL). The molecular docking analyses unveiled the highest binding affinity by *β*-sitosteryl-3*β*-glucopyranoside-6′-*O*-palmitate (**4**) (−8.0 kcal/mol) against *P. aeruginosa* PqsA relative to the ciprofloxacin standard (−8.2 kcal/mol). Furthermore, the organ toxicity predictions showed that all the compounds exhibit no hepatotoxicity and cytotoxicity effects and stigmasterol (**5**) affords drug-likeness protocols. Overall, the combined experimental and computational investigations of this study support the traditional uses of *Ziziphus spina-christi* for antibacterial and natural antioxidant applications.

## 1. Introduction

Nowadays, considerable higher plants are cultivated globally to achieve valuable substances in the fields of medicine and pharmacy. The pharmacological properties of plants gave rise to natural product-derived drugs made from some plants with medicinal advantages. Until the 18^th^ century, the medicinal activities of many plants, their consequence on human health, and their method of action were known. However, the active compounds were unknown [1]. In Ethiopia, medicinal plants have been used in folk prescription against different ailments and their applications in traditional medicine have become an integral part of culture. The traditional health practices and therapies are noted in oral tradition early religious manuscripts and traditional pharmacopoeias [2]. Recent literature sources noticed that about 90% of livestock and 80% of the human population in Ethiopia rely on traditional prescriptions and traditional healers known by different local names are the primary players in the therapeutic aspects of traditional remedy practices [2].

**Figure 1:**
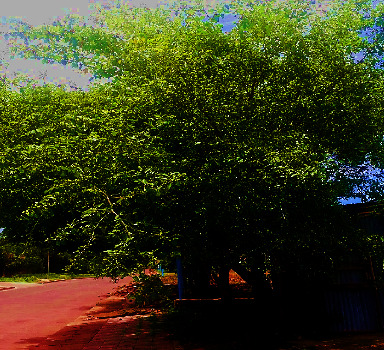
*Ziziphus spina-christi* (picture by Hadush G., October 2022, Adama, Ethiopia).

The genus *Ziziphus* (Rhamnaceae family) is comprised of about 100 species of deciduous trees and shrubs which are cultivated in the tropical and semitropical areas of the world [3]. Among the traditionally useful species of the genus is *Ziziphus spina-christi* (Figure 1). The plant is native to many parts of Africa, including Sudan, Ethiopia, Somalia, Eritrea, Chad, Kenya, Djibouti, Mali, Libya, Mauritania, Senegal, Nigeria, Tunisia, Algeria, and Zimbabwe, and exotic to Saudi Arabia, India, Israel, Comoros, Iran, Egypt, Iraq, Madagascar, Jordan, Morocco, Netherlands, United Arab Emirates, Syrian Arab Republic, and Zanzibar [4]. The traditional applications of *Z. spina-christi* have been studied worldwide for the treatment of numerous ailments [5]. Traditional remedies from the roots, leaves, and flowers of the plant were reported to treat stomach pain in Sudan, Malawi, and Iran [6]. In Bahrain, the extracts of the plant are used against wounds, dandruff, and hair loss [7]. In Turkey and Palestine, the leaves and fruit's fiber contents are used to treat skin infections, and constipation [8, 9]. In Sudan, the fruits are used as remedies for malaria, rheumatism, diarrhea, antispasmodics, and scorpion stings. Decoction is prepared by boiling the fruits and leaves in water for 30 min and then drunk as an oral complement to lower cancer and cholesterol risks [10].

In Ethiopia, *Z. spina-christi* is locally called “Qurqura” (Afan Oromo) and “Gaba” (Amharic and Tigrinya) and possesses substantial traditional uses. In Tigray (North Ethiopia), traditional healers use pounded leaves against dandruff, wounds, and hair loss. In some parts of the region, extracts of the leaves are also used to treat abdominal pain, diarrhea, mastitis, and gastrointestinal complications [11]. Traditional healers around Kunama (North Ethiopia) also use the roots extract mixed with the roots or barks of *Acacia oerfota*, pound together, and mix the fine powder with water and then drink on an empty stomach to treat toothache, stomachache, and tumors [12, 13]. In Oromiya, the fruits have culinary purposes and are used as a remedy against throat infections and hepatitis [14]. The biological activity profiles of *Z. spina-christi* were studied and literature surveys stated that the plant exhibits anti-inflammatory [15], antioxidant [16], antibacterial [17, 18], antimalarial [19], antifungal [20], and anticancer [21, 22] activities with well-documented preparation techniques and mode of actions. Due to its content of tannins, it has significant antibacterial properties. This is because the tannins are linked with the proteins contributing to the metabolic inhibition inside the microorganism and helps to remove it [23]. Treatment of eye infections, headaches, bone pain, colon cramps, scorpion stings, rheumatism, and strengthening the immune system are also other health benefits of the plant [24, 25].

The phytochemical makeup of the plant is broadly diverse, consisting of different classes of natural products mainly terpenoids, saponins, tannins, flavonoids, and alkaloids [26]. Cyclopeptide alkaloids (13–15 member ring polyimides having either basic or neutral side chains) are among the widely reported phytochemicals in the genus *Ziziphus* [10, 27]. Amphibine D, spinanine B and C [10], nummularine U [28], sanjonine B and F, and mauritine A [29] were some cyclopeptide alkaloids reported from various parts of *Z. spina-christi*. The plant also possesses a high composition of saponins in the leaves which affords commercial application in the shampoo and detergent industry [30]. Jujuboside B_1_, dammarane (tetracyclic triterpenes), lotoside III, and christinin were among the reported saponins of the plant [10, 28, 30]. Literature reports also revealed that *Z. spina-christi* is comprised of flavonoids, of which swertisin, spinosin, quercetin-3-O-p-coumaroyl (2,6-dirhamnosyl)-hexoside, quercetin-3-O-[(2-hexonyl)-6-rhamnosyl]-hexoside, kaempferol-3-O-rhamnoside, myricetin-3-O-(6-rhamnosyl) hexoside were frequently reported from various parts of the plant [10, 28, 30, 31]. Terpenoids including ceanothic acid, botulin, betulinic acid, granulosic acid, lupeol acetate, and zizyberanalic acid were also reported from the plant [32]. In addition, the GC-MS analysis revealed that carane, L-menthone, *β*-phellandrene, and *β*-myrcene are major ingredients in the volatile components of *Z. spina-christi* [33].

The recent progress in computational methods has critically introduced the rationale for designing and identifying therapeutically active natural products that can target proteins of interest. The natural molecules isolated from plants can be evaluated by *in silico* approaches to verify their medicinal possibilities [34]. The *in silico* study is a computer-based innovative approach of analysis by enabling research activities without the need of experimental works. In this context, computational analyses have become indispensable in analyzing natural compounds and developing novel drugs. This is because *in silico* study influences the entire drug discovery trajectories with cost and time bargains [35]. *In silico* studies are mainly performed along with *in vitro* investigations, both to verify the potential activities of natural products. In toxicity study, this offers an overview of the use of *in silico* approaches in testing the cytotoxicity of compound isolates and confirms the knowledge about chemical structures and their medicinal implications [36, 37]. Thus, this study was supported by *in silico* analyses to validate the antibacterial and antioxidant properties of isolated natural products from the roots of *Z. spina-christi.*

To the best of our knowledge, there are limited previous phytochemical and biological activity studies on the roots of the plant despite the fact that it is used by Ethiopian traditional healers to cure various infectious diseases. So, inspired by the ethnobotanical profile, phytochemistry, and pharmacological evidence of the plant, this study aims to investigate the *in vitro* antibacterial and antioxidant values of the phytochemicals from the roots extract of *Z. spina-christi* along with their computational studies.

## 2. Materials and Methods

### 2.1. Plant Material

Roots of *Z. spina-christi* were collected from the Adama Science and Technology University campus and its surrounding areas, Adama, Ethiopia, in October 2022. The plant was identified by a taxonomist (Mr. Melaku Wendafrash), and its specimen (HZS007) was deposited at the Herbarium of Addis Ababa University, Ethiopia. The sample materials were washed with distilled water and air-dried for one month at room temperature without direct exposure to sunlight. Finally, the plant material was pounded into fine powder using an electric blender (Shanghai Jinkle, Scientific Instrument, China) and stored in a polyethylene bag until extraction.

### 2.2. Chemicals and Apparatus

*n*-Hexane (99.9%, Pentokey Organy, India), ethyl acetate (99.9%, Sisco Research Laboratories, India), dichloro methane, chloroform, methanol and DMSO (99.8%, Loba Chemie, India), petroleum ether (Indenta Chemicals, India), vanillin (Sisco Research Laboratories, India), ciprofloxacin (Wellona Pharma, India), Mueller Hinton Agar (Micro express, India), DPPH (98.5%, China) Whatman No.1 filter paper (125 mm diameter, India), silica gel for column chromatography (60–200 mesh, Merck, India) and aluminum plate TLC (size; 20 × 20 cm, thickness; 0.25 mm) caked with high-quality silica gel (pore size; 60 Å, grade; 230–400 mesh, Merck Grade 64271, Germany) were obtained from the local markets, Addis Ababa, Ethiopia. All the purchased solvents were in analytical grades.

### 2.3. Instrumentation

Extraction of the plant material (maceration) was performed on an orbital shaker (VRN-200, Taiwan) and the extracts were concentrated in a rotary evaporator (DW-RE-3000, China). The root extracts and fractions were chromatographed on silica gel using column chromatography (column size; 500–725 mm) and eluted with increasing solvent polarity. TLC was used for purity analysis and spots were visualized on a UV lamp (UV4AC6/2, China), sprayed with 1% vanillin-sulphuric acid reagent followed by direct heating at 100°C. Melting points of the compounds were determined on a Japson-type apparatus (JA90161, India). The IR spectral data were generated with the help of a Perkin Elmer Bx infrared spectrometer equipped with KBr pellets and a UV-Vis spectrophotometer (CE4001, Cambridge, UK) was used for antioxidant activity assay. NMR spectral results were performed using 600 MHz Bruker AVANCE III NMR instruments and the residual signals of the solvent (7.28 ppm (*δ*_H_) and 77.2 ppm (*δ*_C_) for CDCl_3_) were used as a reference. For antibacterial activity tests, a hood equipped with UV-radiation and laminar airflow, a micropipette (1000 *µ*L), an incubator, an autoclave, and Petri dishes (90 mm) were utilized.

### 2.4. Extraction and Isolation

The pounded sample of *Z. spina-christi* (450 g) was extracted by maceration technique in 2.25 L (1 g : 5 mL) of CH_2_Cl_2_ : CH_3_OH (1 : 1) followed with an equal volume of CH_3_OH for 3 days each with continuous shaking at room temperature. A Whatman No. 1 type filter paper was employed for filtration of the extracts over gravity filtration, and the filtrates were dried in a rotary evaporator at 40°C under a reduced pressure. Lastly, the extraction yields (%) were calculated using the following equation:

(1) $$\begin{array}{c} \text{Extraction yield}\left(\%\right)=\frac{\text{Weight of the crude extract}}{\text{Weight of the powdered sample}}*100. \end{array}$$

**Scheme 1:**
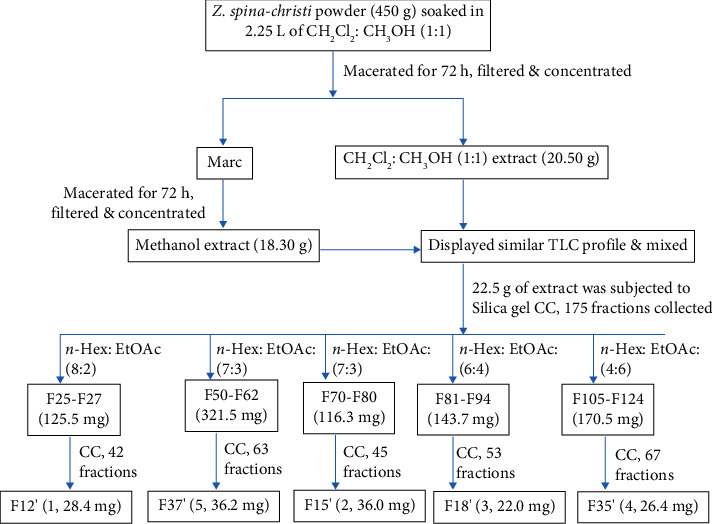
Extraction, isolation, and purification scenario of the roots of *Z. spina-christi*. CC; column chromatography, EtOAc; ethyl acetate, *n*-Hex; *n*-hexane.

Part of the crude extract (22.5 g) was chromatographed on silica gel column chromatography (180 g, 60–200 mesh as stationary phase) and eluted with increasing gradient of ethyl acetate in *n*-hexane followed by methanol in chloroform ratio, successively. A total of 175 fractions (25 mL each) were collected and their purity was monitored with the help of TLC and UV lamp. The retention factor (*R*_*f*_) values of each pure fraction were calculated and fractions with similar *R*_*f*_ values were mixed. Finally, the structural information of the isolated compounds was established following the data obtained from the IR and NMR instruments. Fractions 25–27 obtained with 20% ethyl acetate in *n*-hexane were combined and purified using the gradient elution of *n*-hexane up to 40% ethyl acetate in *n*-hexane to afford trimethyl linolein (**1**) (28.4 mg). In addition, fractions 70–80 isolated with 30% ethyl acetate in *n*-hexane were subjected to gradient elution with 20% ethyl acetate in *n*-hexane up to 60% ethyl acetate in *n*-hexane to yield stearic acid (**2**) (36.0 mg). 13-hydroxyoctadeca-9, 11-dienoic acid (**3**, 22.0 mg) was isolated from fractions 81–94 using the isocratic elution of 40% ethyl acetate in *n*-hexane mixture. Fractions 105–124, collected using 60% ethyl acetate in *n*-hexane were combined and purified with the gradient elution of 20% ethyl acetate in *n*-hexane up to 80% ethyl acetate in *n*-hexane to obtain *β*-sitosteryl-3*β*-glucopyranoside-6′-*O*-palmitate (**4**) (26.4 mg). Furthermore, fractions 50–62 isolated using 30% ethyl acetate in *n*-hexane were mixed and purified with the isocratic elution of 30% ethyl acetate in *n*-hexane as eluents to afford stigmasterol (**5**) (36.2 mg) (Scheme 1).

### 2.5. *In Vitro* Antibacterial Activity Tests

Four bacterial strains namely, *Staphylococcus aureus* (ATCC-25923), *Streptococcus pyogenes* (ATCC-19615), *Escherichia coli* (ATCC-25922), and *Pseudomonas aeruginosa* (ATCC-27853) were attained from the Ethiopian Public Health Institute (EPHI) and the *in vitro* antibacterial activities were performed following previous protocols [38–40] using a commercial ciprofloxacin antibiotic as a positive control. A stock solution of the CH_2_Cl_2_ : MeOH (1 : 1) extract (50 mg/mL) and each compound (2 mg/mL) were prepared in 4% DMSO followed by the preparation of 25 and 12.5 mg/mL (crude extract) and 1 and 0.5 mg/mL (isolated compounds). Each solution (100 *µ*L) was loaded onto the sterilized paper discs using a micropipette and transferred to the Petri dishes comprising the cultured bacterial strains. For complete diffusion, the Petri dishes were left for half an hour and incubated at 37°C for 24 hrs. The measured diameters of inhibition zones (mm) reveal the antibacterial efficiency of the test samples.

### 2.6. *In Vitro* Antioxidant Activity Tests

The antioxidant efficacies were performed using *in vitro* 2, 2-diphenyl-1-picrylhydrazyl (DPPH) radical scavenging assay as per the previous protocol [41] using ascorbic acid and sample-free DPPH as positive and negative controls, respectively. Stock solutions (1000 *μ*g/mL) of the compound isolates, extract, and ascorbic acid standard were prepared in CH_3_OH and were further diluted into four different concentrations (500, 250, 125, and 62.5 *μ*g/mL) using a dilution method. To each prepared concentration, a fresh DPPH solution (1 mL, 0.04% w/v in CH_3_OH) was added. The sample solutions were incubated for 30 min and their absorbances were determined using the UV-Vis spectrophotometer in triplicate at 517 nm. The scavenging potentials were calculated using equation (2) and the results were reported as mean ± SEM. Finally, the IC_50_ values were calculated from the relationship curves.

(2) $$\begin{array}{c} \text{DPPH radical scavenging activity }\left(\%\right)=\left(1-\frac{A}{A_{\circ }}\right)*100, \end{array}$$

where *A* and *A*_∘_ revealed absorbances of the sample and DPPH radical in the prescribed solvent, respectively.

### 2.7. *In Silico* Drug Likeness and Pharmacokinetic Analyses

The drug-likeness predictions were established by adopting Lipiniski's rule of five [42], and the top pharmacokinetic characteristics of the compound isolates were computed using the SwissADME (https://www.swissadme.ch/) online Web tool [43]. Furthermore, the ProTox-II online Web Server (https://tox-new.charite.de/) was applied to establish the organ toxicity properties of the compounds [44].

### 2.8. Molecular Docking Studies

In order to have an understanding of the right binding pocket of the ligands with the protein targets and analyze the observed *in vitro* experimental results [45], two of the compound isolates with promising *in vitro* activities were subjected to molecular docking studies and compared with reference drugs. Molecular docking parameters, such as hydrogen bonds, binding affinity (kcal/mol), and residual amino acid interactions along with 2D and 3D structural depictions of the most stable conformations were provided. Accordingly, the compounds were docked against four bacterial protein targets namely, *S. aureus* PK (PDB ID: 3T07), *S. pyogenes* 10782 streptopain (PDB ID: 6UKD), DNA gyrase B of *E. coli* (PDB ID: 6F86), and PqsA of *P. aeruginosa* (PDB ID: 5OE3) and a single human enzyme namely, human myeloperoxidase (PDB ID: 1DNU). The bacterial protein targets were chosen based on their metabolic significance to the bacteria and the similarity of the compound isolates to the co-crystallized ligands in the protein complexes.

#### 2.8.1. Protein Preparation

The protein preparations were performed following the reported standard protocol [46]. Initially, the crystal structures of the protein targets were retrieved from the Protein Data Bank (https://www.rcsb.org) using their PDB identifications. The 3D crystal structures were imported into Biovia Discovery Studio Visualizer 2021 followed by the removal of water molecules, heteroatoms, and complexes bound to the receptor molecules. To compute a site-specific molecular docking, ligand groups were selected from the co-crystallized protein structures and defined, and the binding sites were retrieved by identifying the *x*, *y*, and *z* coordinates from the SBD site spheres. The active sites were recognized by studying the binding interaction of the ligand and the receptor and the dockings were computed on the active sites of the prepared receptors. Thereby, the dimensions of the SBD site sphere of each target protein were established as follows: PDB ID: 3T07 (0.014, 0.147, and −0.003), 6UKD (−25.515, −13.743, and 13.171), 6F86 (61.680, 28.330, and 64.290), 5OE3 (29.262, 21.848, and 19.863), and 1DNU (36.727, 9.922, and 10.989) Å for *x*, *y* and *z* coordinates, respectively. Subsequently, polar hydrogens were added for the correct ionization of the amino acid residues [47, 48]. Finally, the energy of the proteins was minimized using the CHARMM Force Field steepest descent algorithm with a maximum number of 1000 steps at a root mean square (RMS) gradient of 0.01. The energy minimization process was continued until the protein fulfilled a convergence gradient of 0.001 kcal·mol^−1^ [46].

#### 2.8.2. Ligand Preparation

The structures of the compound isolates were drawn on the ChemOffice tool (Chem Draw 16.0) and assigned with suitable 2D orientations. By applying the ChemBio3D tool, energy minimizations were performed with the molecular modeling (MM2) algorithm until it fulfilled the RMS gradient of 0.01 kcal·mol^−1^ to achieve the minimum energy conformers. Lastly, the ligand molecules with minimized energy were saved in PDB format and used as an input for the AutoDock Vina to perform molecular docking simulations [46].

#### 2.8.3. Protein-Ligand Docking Strategy

The docking protocol was validated to confirm the reliability and accuracy of the molecular docking outcomes, as per previous protocols [46, 49, 50]. The objective was to precisely reproduce the docking interactions and binding pores of the co-crystallized ligands inside the experimentally crystallized structure of proteins. Thus, the original ligand of the co-crystallized protein was separated and prepared for the molecular docking of the ligands of interest. The ligands of interest were then docked back into the active sites of the protein using the AutoDock Vina 4.2 software, and all the saved files were run via Command Prompt to generate the affinity and root mean square deviation (RMSD) values [47]. Notably, the RMSD values ranging from (0–2) Å were suitable for the molecular docking demonstrating that the protocol is feasible for other compound inhibitors [50]. For each ligand scored, nine different poses were generated and the conformation of the most stable binding affinity and RMSD (small binding scores) was preferred to study the interactions between the ligands and receptor. Finally, the image preparations, ligand interactions, and orientations were computed using the Biovia Discovery Studio Visualizer 2021 [51–53].

### 2.9. Spectral and Statistical Data Analysis

The IR and NMR data were manipulated using Origin 8.0 and MestReNova softwares, respectively. The *in vitro* antioxidant and antibacterial data were tabulated in a Microsoft Excel 2013 spreadsheet, and the values were reported as percent of scavenging activity and mean ± standard error of the mean (SEM), respectively. The antibacterial activities were deduced by the relative comparison of the inhibition zones of the compounds and extract with the standard antibiotic (ciprofloxacin).

## 3. Results and Discussion

### 3.1. Extraction Yield

The yields of the CH_2_Cl_2_ : CH_3_OH (1 : 1) and CH_3_OH roots extracts of *Z. spina-christi* were calculated using equation (1) to afford 20.50 g (4.55%) and 18.30 g (4.07%) of crude samples for the respective extracts. The extracts displayed a similar TLC profile and were hence mixed to yield 38.80 g.

### 3.2. Structure Elucidations

Five compounds, namely, trimethyl trilinolein (**1**), stearic acid (**2**), 13-hydroxyoctadeca-9, 11-dienoic acid (**3**), *β*-sitosteryl-3*β*-glucopyranoside-6′-*O*-palmitate (**4**), and stigmasterol (**5**) were isolated from the CH_2_Cl_2_ : CH_3_OH (1 : 1) and CH_3_OH extracts of *Z. spina-christi.* The compounds were characterized with the help of IR and NMR spectroscopic techniques and their structural information was supported by literature references.

Compound **1** (28.40 mg, *R*_*f*_: 0.83 in 20% ethyl acetate in *n*-hexane) was isolated as a pale yellow substance. The FTIR spectrum revealed strong absorption bands at 2918 and 2849 cm^−1^ attributed to -C-H stretching frequencies in the aliphatic region. In addition, the strong and weak absorption bands observed at 1739 and 1162 cm^−1^ are evident to the presence of -C=O and -C-O-C stretching frequencies in esters, respectively (Supplementary Materials, Table S1, and Figure S1). The ^1^H NMR (600 MHz, CDCl_3_) spectrum showed signals at *δ* 5.40 (3H, *m*, H-10′, 10″), 5.38 (3H, *m*, H-9′, 9″), and 5.14 (3H, *t*, *J* = 7.4, H-13′, 13″) suggesting the presence of three groups of olefinic protons. The multiplet peaks observed at *δ* 5.29 (1H, *m*, H-2) and 4.32 (2H, *dd*, *J* = 12.0, 4.4, H-1b, 3b), 4.17 (2H, *dd*, *J* = 11.8, 5.8, H-1a, 3a) attributed to oxymethine and oxymethylene protons of a glycerol backbone in triglycerides, respectively. The doublet and triplet signals at *δ* 2.79 (6H, *d*, *J* = 6.9, H-11′, 11″) and 2.33 (6H, *t*, *J* = 7.6, H-2′, 2″) correspond to the methylene protons flanked between the unsaturation and those adjacent to the carbonyl groups, respectively. Furthermore, the multiplet signals at *δ* 2.07 (12H, *m*) and 1.63 (6H, *m*) were assignable to (H-8′, 8″, 14′, 14″) and (H-3′, 3″), respectively. The spectrum also exhibited signals of methyl protons at *δ* 1.70 (9H, *s*, H-19′, 19″) and 0.90 (9H, *brt*, *J* = 6.7, H-18′, 18″). The remaining protons appeared as multiplet signals at *δ* 1.33 (6H, *m*, H-17′, H-17″) and 1.28 (36H, *m*, H-4′, H-4″, H-5′, H-5″, H-6′, H-6″, H-7′, H-7″, H-15′, H-15″, H-16′, and H-16″) (Supplementary Materials, Table S2, and Figure S2).

**Figure 2:**
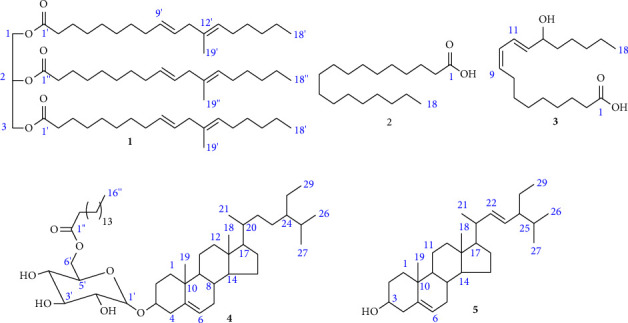
Structural information of the compound isolates (**1–5**) from *Z. spina-christi* (roots).

Notably, the ^13^C NMR (150 MHz, CDCl_3_) spectrum displayed carbonyl signals at *δ* 173.2 (C-1′) and 172.1 (C-1″), olefinic carbons at *δ* 135.2 (C-12′, 12″), 130.2 (C-9′, 9″), 127.9 (C-10′, 10″), and 125.0 (C-13′, 13″), oxymethine and oxymethylene signals at *δ* 68.9 (C-2) and 62.1 (C-1, 3), respectively, associated to the structure of triglycerides with unsaturated fatty acid chains. The presence of methyl signals at *δ* 23.4 (C-19′, C-19″) and a quaternary carbon at *δ* 135.2 (from DEPT-135 spectrum) are evident that one of the olefinic moiety of the fatty acid group exhibits an additional methyl substituent (Supplementary Materials, Table S2, and Figure S3). In addition, the types of carbons were verified by the DEPT-135 spectrum which revealed three olefinic signals assignable to nine carbons, one oxymethine, and two oxymethylene signals, twelve sp^3^ methylene signals assignable to thirty-six carbons, and two methyl signals assignable to six carbons in accordance with the ^13^C NMR spectrum of the compound (Supplementary Materials, Table S2, and Figure S4). Finally, the spectral data agreed with the literature values [54, 55] of trimethyl trilinolein (**1**) (Figure 2).

Compound **2** (36.00 mg, *R*_*f*_: 0.63 in 20% ethyl acetate in *n*-hexane, mp: 69–71°C) was obtained as white crystals. It was UV inactive and a vanillin-sulphuric acid spray was applied on the TLC plate followed by heating on a hot plate at 110°C for visualization. In its FTIR spectrum, a broad absorption band observed between 3500 and 2554 cm^−1^ suggested the presence of a carboxylic acid functional group. In addition, the strong absorption bands at 2918 and 2842 cm^−1^ correspond to the -C-H stretching frequencies in the aliphatic region. The spectrum also showed absorption bands at 1705 and 1167 cm^−1^ attributed to -C=O and –C-O stretching frequency of carboxylic acids, respectively (Supplementary Materials, Table S3, and Figure S5).

The ^1^H NMR (600 MHz, CDCl_3_) spectrum of the compound displayed triplet signals at *δ* 2.37 (2H, *t*, *J* = 7.6) and 0.90 (3H, *t*, *J* = 7.0) attributed to the methylene protons at C-2 and terminal methyl protons (C-18) of a fatty acid skeleton, respectively. The spectrum also exhibited multiplet peaks at *δ* 1.66 (2H, *m*) and 1.28–1.32 (26H, *m*) assignable to C-3, and C-4 up to C-17 of the fatty acid chain, respectively (Supplementary Materials, Table S4, and Figure S6). In its ^13^C NMR (150 MHz, CDCl_3_) spectrum, the compound displayed eleven signals assignable to eighteen carbons. The signals observed at *δ* 179.3 (C-1) and 14.1 (C-18) revealed the presence of carbonyl and terminal methyl carbons of a fatty acid skeleton. Additional signals observed at *δ* 33.9, 31.9, 29.6, 29.4, 29.3, 29.2, 29.0, 24.6, and 22.7 are assignable to C-2, C-16, (C-6 up to C-13), C-14, C-15, C-5, C-4, C-3, and C-17, respectively (Supplementary Materials, Table S4, and Figure S7). Notably, the DEPT-135 spectrum revealed one methyl signal at *δ* 14.1 and sixteen methylene signals which were consistent with the ^13^C NMR spectral analysis of the compound (Supplementary Materials, Table S4, and Figure S8). Overall, the data generated match with previous work of Abdurrahman and Cai-Xiab [56] for stearic acid (**2**) (Figure 2). The compound was also identified from the GC-MS analysis of the volatile oils of *Z. spina-christi* and its structural information was supported by mass fragmentation patterns (Supplementary Materials, Figure S9).

Compound **3** (23.00 mg, *R*_*f*_: 0.49 in 30% ethyl acetate in *n*-hexane) was obtained as a yellow powder and its ^1^H NMR (600 MHz, CDCl_3_) spectrum showed signals at *δ* 6.51 (1H, *dd*, *J* = 15.2, 11.1, H-11), 5.99 (1H, *t*, *J* = 11.0, H-10), 5.68 (1H, *dd*, *J* = 15.2, 6.9, H-12), and 5.48 (1H, *m*, H-9) suggesting the presence of four olefinic protons. It also displayed signals of oxymethine and hydroxyl protons at *δ* 4.18 (1H, *q*, *J* = 6.6, H-13) and 3.34 (1H, *s*) evident for the presence of a secondary alcohol. The triplet and multiplet peaks at *δ* 2.37 (2H, *t*, *J* = 7.5) and 2.20 (2H, *m*) were assignable to the methylene protons adjacent to the carbonyl (H-2) and olefinic protons (H-8), respectively. Furthermore, the triplet signals observed at *δ* 0.91 (3H, *t*, *J* = 7.3, H-18) attributed to the terminal methyl protons of a fatty acid chain. The remaining protons overlapped in the range of *δ* 1.50–1.62 (4H, *m*) and 1.28–1.34 (14H, *m*) (Supplementary Materials, Table S5, and Figure S10).

The ^13^C NMR (150 MHz, CDCl_3_) spectrum revealed the resonance of eighteen carbons associated with the structure of linoleic acid. This was confirmed by the presence of a carbonyl signal at *δ* 179.7, olefinic signals at *δ* 135.6 (C-12), 133.1 (C-9), 127.6 (C-10), and 125.9 (C-11), and a terminal methyl signal at *δ* 14.1 (C-18). The signal at *δ* 72.9 demonstrated an oxymethine carbon assignable to C-13. Additional aliphatic signals observed at *δ* 37.2, 33.7, 31.9, 31.4, 29.7, 29.3, 29.1, 27.7, 25.3, 24.6, 22.7, and 14.1 are assignable to C-14, C-2, C-16, C-7, C-6, C-5, C-4, C-8, C-15, C-3, C-17, and C-18, respectively (Supplementary Materials, Table S5, and Figure S11). In addition, the types of carbons were identified by the DEPT-135 spectral data. Thereby, four sp^2^ olefinic carbons, one oxymethine carbon, eleven sp^3^ methylene signals, and one methyl signal were displayed in the spectrum which agreed with the ^13^C spectral data of the compound (Supplementary Materials, Table S5, and Figure S12). Overall, the spectral analyses match with the reported values of d'Almeida Gameiro and co-workers [57] for 13-hydroxyoctadeca-9, 11-dienoic acid (**3**) (Figure 2).

Compound **4** (26.42 mg, *R*_*f*_: 0.43 in 40% ethyl acetate in *n*-hexane) was isolated as white crystals and its ^1^H NMR (600 MHz, CDCl_3_) spectrum exhibited multiplet signals at *δ* 5.34 (1H, *m*, H-6) signifying the presence of a single olefinic proton. A group of signals observed at *δ* 4.37 (1H, *dd*, *J* = 12.1, 5.2, H-6′a), 4.27 (1H, *d*, *J* = 11.5, H-1′), 4.13 (1H, *dd*, *J* = 12.1, 6.3, H-6′b), 3.65 (1H, *m*, H-3′), 3.37 (1H, *m*, H-4′), 3.44 (1H, *m*, H-5′), and 3.36 (1H, *m*, H-2′) suggest the presence of a glucose moiety. The signals observed at *δ* 3.54 (1H, *m*, H-3) were assignable to an oxymethine proton evidencing the presence of a glycoside linkage. In addition, the singlet peaks appeared in the range *δ* 0.66–0.99 (18H, *s*, H-18, H-19, H-21, H-26, H-27, and H-29) confirm the presence of a steroidal skeleton that corresponds to the methyl groups of the main skeleton. The attachment of an extra fatty acid moiety was confirmed by the appearance of long-chain methylene signals in the upfield region of both the ^1^H and ^13^C NMR spectra. The majority of the proton signals overlaid in the region *δ* 2.40–1.00 (Supplementary Materials, Table S6, and Figure S13).

The ^13^C NMR (150 MHz, CDCl_3_) spectrum revealed forty-five well-resolved carbon signals assignable to fifty-one carbons attributed to a steroidal skeleton with additional glucose and fatty acid moieties. The fatty acid moiety was confirmed by the presence of an ester carbonyl at *δ* 174.7 (C-1″) and the type of the fatty acid chain was verified as palmitic acid based on the acid-catalyzed methanolysis followed by GC-MS analysis. The signals at *δ* 140.3 and 122.2 assignable to C-5 and C-6, respectively, indicated the presence of two olefinic carbons of which the first *δ* value corresponds to an sp^2^ quaternary carbon (also supported by DEPT-135 spectrum). The spectrum also showed the resonance of six sugar signals at *δ* 101.2 (C-1′), 76.1 (C-3′), 73.9 (C-5′), 73.6 (C-2′), 70.1 (C-4′), and 63.4 (C-6′) of which the signal of the anomeric carbon resonates at *δ* 101.2. Another oxymethine signal observed at *δ* 79.7 attributed to the carbon atom forming a glycoside linkage (C-3) in the main skeleton of the structure. In addition, the carbon signals at *δ* 56.8, 56.1, 50.2, 45.8, and 36.2 correspond to the sp^3^ methine carbons of the structure and were assignable to C-14, C-17, C-9, C-24, and C-20, respectively. The spectrum also showed two sp^3^ quaternary carbons at *δ* 42.4 (C-13) and 36.8 (C-10) supported by the DEPT-135 spectrum. The values of the remaining methylene and methyl signals are clearly stated in the supporting information (Supplementary Materials, Table S6, and Figure S14). In agreement with the ^13^C NMR spectral data, its DEPT-135 spectrum displayed one sp^2^ methine, six oxymethines, one oxymethylene, seven sp^3^ methines, nineteen sp^3^ methylenes assignable to twenty-five carbons, and seven methyl signals (Supplementary Materials, Table S6 and Figure S15). Moreover, the COSY spectrum showed correlations for two spin systems. Accordingly, ^3^*J* correlations were observed between H-2″ (*δ* 2.31) and H-3″ (*δ* 1.61) (Supplementary Materials, Table S6, and Figure S16). Finally, the data generated match with the reported values [58] of *β*-sitosteryl-3*β*-glucopyranoside-6′-*O*-palmitate (**4**) (Figure 2).

Compound **5** (36.20 mg, mp: 167-168°C, *R*_*f*_: 0.75 in 25% ethyl acetate in *n*-hexane) was isolated as yellow-green solid crystals. The melting point matches with the reported values of Amaro-Luis and co-workers [59] for stigmasterol (165–168°C). The ^1^H NMR (600 MHz, CDCl_3_) spectrum displayed signals at *δ* 5.35 (1H, *dd*, *J* = 5.0, 2.7, H-6), 5.14 (1H, *dd*, *J* = 15.1, 8.6, H-22), and 5.00 (1H, *dd*, *J* = 15.2, 8.6, H-23) signifying the presence of three olefinic protons. The spectrum also displayed multiplet peaks at *δ* 3.51 (1H, *m*, H-3) assignable to oxymethine protons and the presence of six groups of methyl protons observed at *δ* 1.02 (3H, *d*, *J* = 7.2, H-21), 0.99 (3H, *s*, H-19), 0.83 (3H, *d*, *J* = 6.1, H-27), 0.82 (3H, *d*, *J* = 6.3, H-26), 0.79 (3H, *t*, *J* = 6.0, H-29), and 0.68 (3H, *s*, H-18) was evident for a stigmastane tetracyclic triterpenoid. The majority of the proton signals overlapped in the range between *δ* 2.30 and 0.90 (25H, *m*) (Supplementary Materials, Table S7, and Figure S17).

The ^13^C NMR (150 MHz, CDCl_3_) spectrum displayed twenty-nine carbons associated with the structure of stigmastane tetracyclic triterpenoids. The signals at *δ* 140.8 (C-5), 138.4 (C-22), 129.3 (C-23), and 121.8 (C-6) were assignable to the olefinic carbons and the signal at *δ* 71.9 attributed to the oxymethine carbon of the compound (C-3). The spectrum also displayed signals at *δ* 56.9 (C-14), 56.0 (C-17), 51.3 (C-24), and 50.2 (C-9) conforming to the sp^3^ methine signals of the structure. Furthermore, aliphatic carbon signals were observed at *δ* 42.4, 42.3, 40.6, 39.7, 37.3, 36.6, 32.0, 31.9 (x 2), 31.7, 29.8, 25.5, 24.4, 21.3, 21.2, 21.1, 19.5, 19.0, 12.3, and 12.1 assignable to C-4, C-13, C-20, C-12, C-1, C-10, C-7, (C-8, 25), C-2, C-16, C-28, C-15, C-26, C-21, C-11, C-19, C-27, C-29, and C-18, respectively (Supplementary materials, Table S7, and Figure S18). In agreement with the ^13^C NMR spectral data, the DEPT-135 spectrum revealed three sp^2^ olefinic carbons, one oxymethine, seven methine, six methyl signals pointing up, and nine methylene signals pointing down (Supplementary Materials, Table S7, and Figure S19).

The ^1^H NMR assignments were verified by 2D COSY correlations. Accordingly, the spectrum revealed ^3^*J* correlations between H-1b (*δ* 1.09) and H-2a (*δ* 1.82), H-3 (*δ* 3.51) and H-4a (*δ* 2.25), H-6 (*δ* 5.35) and H-7a (*δ* 1.96), H-7a (*δ* 1.96) and H-8 (*δ* 1.49), H-15 (*δ* 1.52) and H-16 (*δ* 1.81), H-20 (*δ* 2.03) and H-21 (*δ* 1.02), H-22 (*δ* 5.14) and H-23 (*δ* 5.00), and H-25 (*δ* 1.43) and H-26, 27 (*δ* 0.82, 0.83) (Supplementary Materials, Table S7, and Figure S20). Lastly, the spectral analyses agreed with the literature values [60] of stigmasterol (**5**) (Figure 2).

### 3.3. Antibacterial Activity

The agar disc diffusion evaluation results revealed that the crude extract and isolated compounds exhibited auspicious antibacterial activities against the bacterial strains. The choice of bacterial pathogens was based on their prevalence and availability in Ethiopia. The average zones of inhibition (mm) against the growth of the bacterial pathogens are presented in Table 1 and the activities showed smooth relationships with concentration. Accordingly, the crude extract and isolated compounds displayed better activities at 50 and 2 mg/mL, respectively. For the CH_2_Cl_2_ : CH_3_OH (1 : 1) extract, the highest inhibition zone (15.25 ± 0.35 mm) was observed at 50 mg/mL against *E. coli* compared to ciprofloxacin (21.00 ± 0.35 mm) and the lowest activity (9.50 ± 0.70 mm) was displayed at 12.5 mg/mL against *P. aeruginosa*. Our results align with previous reports against different bacterial strains. Previous work on methanol extracts of the roots and leaves of *Z. spina-christi* revealed promising activities against *E. coli* (15.00 ± 0.50 mm) and *P. aeruginosa* (12.00 ± 1.00 mm), respectively [61]. In a related study by Ads et al. [32], the chloroform extract from the stem bark of *Z. spina-christi* displayed substantial antibacterial efficacy against *E. coli* (15.90 ± 0.63 mm), *S. aureus* (16.40 ± 1.20 mm), and *P. aeruginosa* (15.30 ± 1.50 mm) and the activities match with the results of the present work.

Our study also evaluated the activities of five isolated compounds (**1–5**) against the four bacterial strains and the results were attractive. The strongest activity was observed for *β*-sitosteryl-3*β*-glucopyranoside-6′-*O*-palmitate (**4**) (14.25 ± 0.35 mm) against *E. coli* compared to ciprofloxacin (21.00 ± 0.35 mm) at 2 mg/mL. Furthermore, at the smallest concentration (0.5 mg/mL), *β*-sitosteryl-3*β*-glucopyranoside-6′-*O*-palmitate (**4**) also exhibited better activity (9.25 ± 0.35 mm) against *E. coli* and *P. aeruginosa* followed by stigmasterol (**5**) against *S. aureus* (9.00 ± 0.70 mm) (Table 1). Generally, the antibacterial activities of the extract and compounds support the ethno-medicinal uses of *Z. spina-christi*.

### 3.4. Antioxidant Activity

The CH_2_Cl_2_ : CH_3_OH (1 : 1) extract and compound isolates (**1–5**) were subjected to *in vitro* antioxidant activities and the results were auspicious. At 1000 *µ*g/mL, the CH_2_Cl_2_ : CH_3_OH (1 : 1) extract exhibited promising scavenging activity (91.81 ± 0.28%) and IC_50_ value (1.51 *µ*g/mL) compared to ascorbic acid (98.30 ± 0.00%). Literature surveys showed that the smaller the IC_50_ value, the better DPPH radical scavenging activity [48, 62]. Of the compound isolates, *β*-sitosteryl-3*β*-glucopyranoside-6′-*O*-palmitate (**4**) showed better-scavenging potential (IC_50_ : 5.41 *µ*g/mL) than the others followed by stigmasterol (**5**) (IC_50_ : 6.88 *µ*g/mL) and trimethyl trilinolein (IC_50_ : 7.67 *µ*g/mL) (Table 2) compared to ascorbic acid (IC_50_ : 0.46 *µ*g/mL). In general, the antioxidant potentials can be related to the ability of compounds to trap free radicals and inhibit oxidation [48], thereby *β*-sitosteryl-3*β*-glucopyranoside-6′-*O*-palmitate (**4**) has many proton donating groups and hence displayed greater scavenging activity.

**Figure 3:**
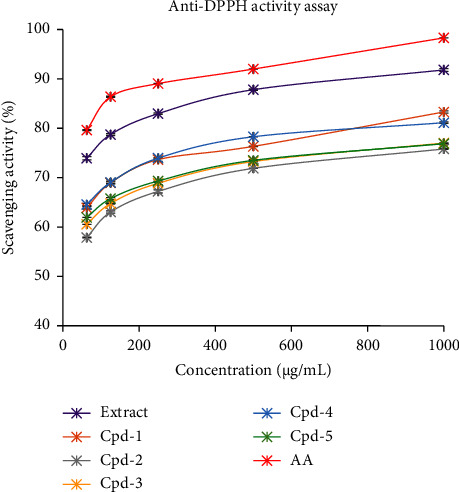
Radical (DPPH) scavenging activity (%) of CH_2_Cl_2_: CH_3_OH (1 : 1) extract, compound isolates (**1**–**5**), and ascorbic acid standard (graphical depiction). AA; ascorbic acid.

A Microsoft Excel 2016 spreadsheet was used to calculate the IC_50_ values. The logarithmic relationship curves that displayed the closest coefficient of determination (*R*^2^) values to one were chosen and the following equations were set. CH_2_Cl_2_ : CH_3_OH (1 : 1) extract; *Y* = 6.46ln(*x*) + 47.33, *R*^2^ = 0.99, trimethyl trilinolein (**1**); *Y* = 6.66ln(*x*) + 36.41, *R*^2^ = 0.98, stearic acid (**2**); *Y* = 6.45ln(*x*) + 31.54, *R*^2^ = 0.99, 13-hydroxyoctadeca-9, 11-dienoic acid (**3**); *Y* = 5.97ln(*x*) + 35.89, *R*^2^ = 0.99, *β*-sitosteryl-3*β*-glucopyranoside-6′-*O*-palmitate (**4**); *Y* = 6.11ln(*x*) + 39.63, *R*^2^ = 0.99, stigmasterol (**5**); *Y* = 5.42ln(*x*) + 39.52, *R*^2^ = 0.99, and ascorbic acid; *Y* = 6.19ln(*x*) + 54.84, *R*^2^ = 0.97, and the *Y* values were labeled as 50 (Figure 3).

### 3.5. *In Silico* Pharmacokinetic Predictions

In the present study, Lipinski's rule of five [42] and Veber's rule [63] were applied to predict the drug-likeness properties of the isolated compounds. Compounds with MW < 500 Daltons, NHA < 10, NHD < 5, and LogP (iLogP) <5 satisfy Lipinski's rule of 5 and are good drug candidates. In addition, predicted compounds with TPSA < 140 and NRB < 10 satisfy Veber's rule and afford good oral bioavailability [47, 48]. Generally, therapeutic agents with high molecular weight (>500 Daltons) decrease absorption by reducing their concentrations in the intestinal epithelial surface leading to passive diffusion across the bilayer membrane [64, 65]. Thus, drug candidates with MW < 500 Daltons are suggested in accordance with Lipiniski's rule of five. Three of the investigated compounds in this study exhibited molecular weight within the recommended range of drug-like compounds as presented in Table 3. Compounds with zero or one violation are chosen for drug candidacy, thereby 13-hydroxyoctadeca-9, 11-dienoic acid (**3**) satisfied Lipinski's rule with no violation. Fewer violations (1) were also predicted for stearic acid (**2**), and stigmasterol (**5**) which afford the concept of drug candidacy. Only stigmasterol (**5**) obeyed Veber's rule with NRB = 5 and TPSA = 20.23 Å^2^ and hence safe absorption by the gut. The LogP value provides information on the affinity of compounds to lipophilic properties [66]. Thus, stearic acid (**2**) and 13-hydroxyoctadeca-9, 11-dienoic acid (**3**) exhibited <5 values of LogP suggesting their optimum lipophilicity (Table 3). The ADME properties of the isolated compounds along with ciprofloxacin are also portrayed in Table 3. The LogKp (cm/s) value of 13-hydroxyoctadeca-9, 11-dienoic acid (**3**) (−4.19) was predicted better than the others relative to ciprofloxacin (−9.09). The smaller the LogKp (cm/s), the lower the skin permeability of the compound [43]. Trimethyl trilinolein (**1**) revealed a noninhibitor of all the cytochromes and was in accordance with the prediction results of ciprofloxacin (Table 3).

According to Gadaleta et al. [67] toxicity predictions, trimethyl trilinolein (**1**) showed LD_50_ >5000 mg/kg and hence no toxicity. Notably, stearic acid (**2**), 13-hydroxyoctadeca-9, 11-dienoic acid (**3**), *β*-sitosteryl-3*β*-glucopyranoside-6′-*O*-palmitate (**4**), and stigmasterol (**5**) displayed LD_50_ values in the range 500 < LD_50_ ≤ 5000 mg/kg with toxicity classes of 4 and 5, and were slightly toxic. The toxicity predictions also showed that stearic acid (**2**) and 13-hydroxyoctadeca-9, 11-dienoic acid (**3**) were inactive towards all the inhibitor interactions, trimethyl trilinolein (**1**) was active only towards carcinogenicity and *β*-sitosteryl-3*β*-glucopyranoside-6′-*O*-palmitate (**4**) and stigmasterol (**5**) were active only towards immunotoxicity (Table 4). Overall, the compounds of study with special emphasis to stigmasterol (**5**), can be good therapeutic agents, and we recommend further biological and computational studies.

**Figure 4:**
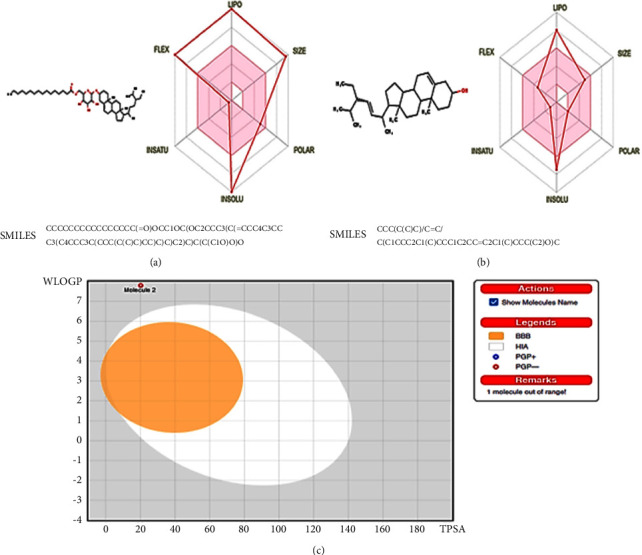
Bioavailability radar (a and b) and BOILED-Egg model (c) of *β*-sitosteryl-3*β*-glucopyranoside-6′-*O*-palmitate (**4**) and stigmasterol (**5**). Molecule 1; *β*-sitosteryl-3*β*-glucopyranoside-6′-*O*-palmitate (**4**), and molecule 2; stigmasterol (**5**).

In this study, the drug-likeness properties of *β*-sitosteryl-3*β*-glucopyranoside-6′-*O*-palmitate (**4**) and stigmasterol (**5**) were supported by BOILED-Egg model and bioavailability radar predictions. The insolubility (INSOLU), molecular weight (SIZE), optimal area for unsaturation (INSATU), polar surface area (POLAR), rotatable bonds (FLEX), and lipophilicity (LIPO) properties of the two compounds were portrayed in the bioavailability radar (Figure 4). Drug-like molecules are proposed to unveil −0.7 < LIPO (iLog P) < 5, SIZE (MW) < 500 g/mol, 20 Å^2^ < POLAR (TPSA) < 140 Å^2^, −6 < INSOLU (Log S) < 0, 0.25 < INSATU (fraction Csp3) < 1, and 0 < FLEX (NRB) < 9 predictions [47, 68]. Accordingly, stigmasterol (**5**) with SIZE = 412.69 g/mol, TPSA = 20.23 Å^2^, fraction Csp3 = 0.86, and NRB = 5 showed auspicious drug-like properties than *β*-sitosteryl-3*β*-glucopyranoside-6′-*O*-palmitate (**4**) which favored only the POLAR and INSATU values of the bioavailability radar. In addition, a BOILED-Egg model was used to establish the pharmacokinetic properties of the two compounds and to explain the blood-brain barrier (BBB) and human intestinal absorption (HIA) features. Compounds with high HIA and BBB penetration are located in the albumin (white) and yolk (yellow) regions, respectively [47, 68]. Neither *β*-sitosteryl-3*β*-glucopyranoside-6′-*O*-palmitate (**4**) nor stigmasterol (**5**) was located inside the prescribed regions explaining their poor penetration and absorption. Stigmasterol (**5**) was also marked along with a red circle, suggesting its nonsubstrate (PGP-) properties (Figure 4). Hence, the BOILED-Egg model and bioavailability radar predictions suggest more drug-like capabilities for stigmasterol (**5**).

### 3.6. Molecular Docking Studies

In the present work, *in silico* molecular docking studies aimed to consolidate the antibacterial activities were computed towards selected protein targets of the bacterial strains and the results match the *in vitro* experimental findings. Accordingly, *β*-sitosteryl-3*β*-glucopyranoside-6′-*O*-palmitate (**4**) and stigmasterol (**5**) which showed promising antibacterial activities were the subjects of the study. The compounds exhibited binding affinities of −7.3 (**4**) and −7.0 kcal/mol (**5**) against *E. coli* DNA gyrase B and the scores were closer to ciprofloxacin (−7.6 kcal/mol). The molecular docking scores of *β*-sitosteryl-3*β*-glucopyranoside-6′-*O*-palmitate (**4**) and stigmasterol (**5**) against *P. aeruginosa* PqsA showed binding affinities of −8.0 and −7.8 kcal/mol, respectively, and the results were comparable to ciprofloxacin (−8.2 kcal/mol). In this regard, *β*-sitosteryl-3*β*-glucopyranoside-6′-*O*-palmitate (**4**) displayed better docking scores than stigmasterol (**5**) against the two protein targets. Conversely, stigmasterol (**5**) showed higher scores (−7.7 and −7.4 kcal/mol) than *β*-sitosteryl-3*β*-glucopyranoside-6′-*O*-palmitate (**4**) (−7.6 and −5.9 kcal/mol) against *S. aureus* PK and *S. pyogenes* 10782 streptopain protein targets, respectively. The results were also not far from the docking scores of ciprofloxacin (−7.9 and −7.6 kcal/mol against *S. aureus* PK and *S. pyogenes* 10782 streptopain, respectively) (Table 5). Streptopain protein is a key virulence factor in streptococcal exposures causing different human ailments including fever, pharyngitis, glomerulonephritis, and shock-like syndrome [47, 69], thereby it was targeted in the *in silico* docking study of this work.

**Figure 5:**
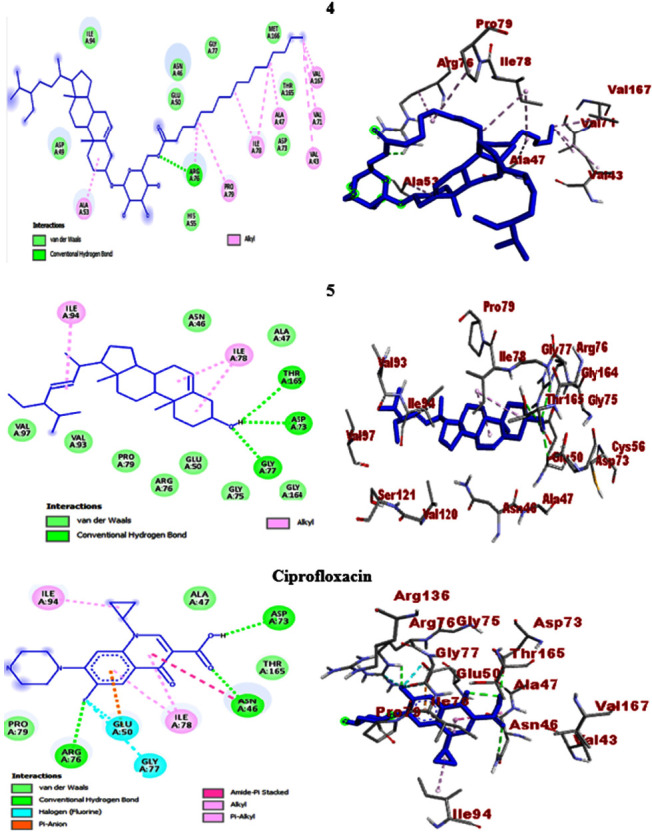
2D (left) and 3D (right) binding interactions of compounds **4**, **5**, and ciprofloxacin against *E. coli* DNA gyrase B.

**Figure 6:**
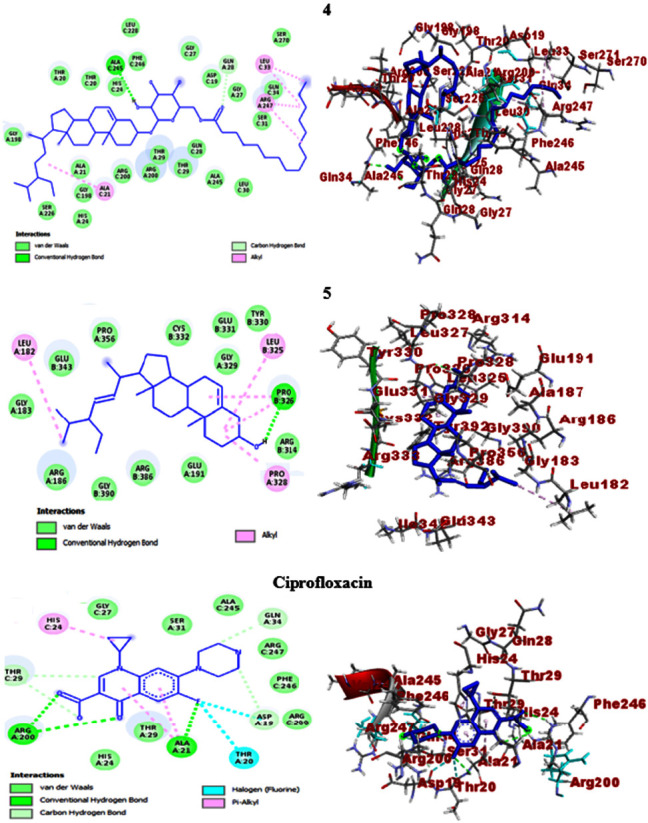
2D (left) and 3D (right) binding interactions of compounds **4**, **5**, and ciprofloxacin against *P. aeruginosa* PqsA.

**Figure 7:**
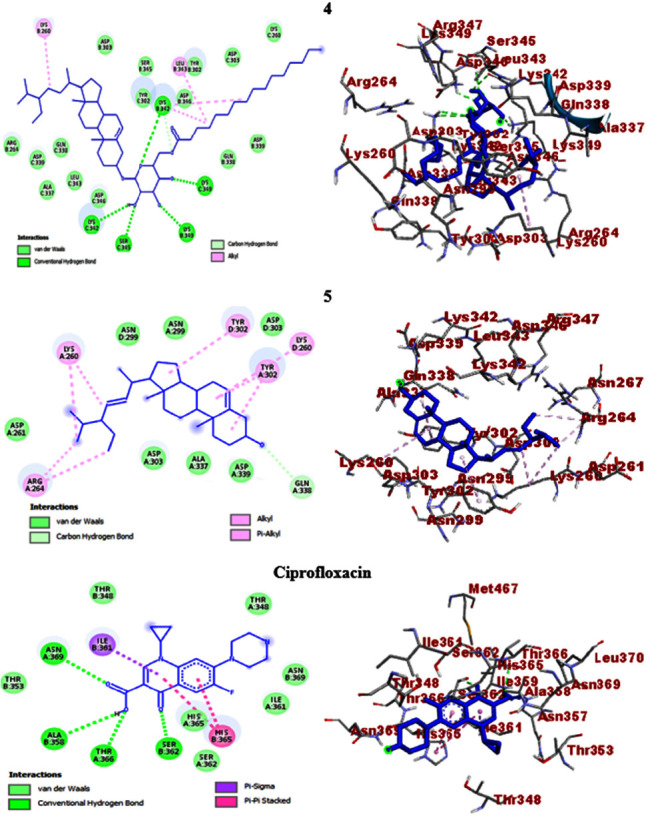
2D (left) and 3D (right) binding interactions of compounds **4**, **5**, and ciprofloxacin against *S. aureus* PK.

**Figure 8:**
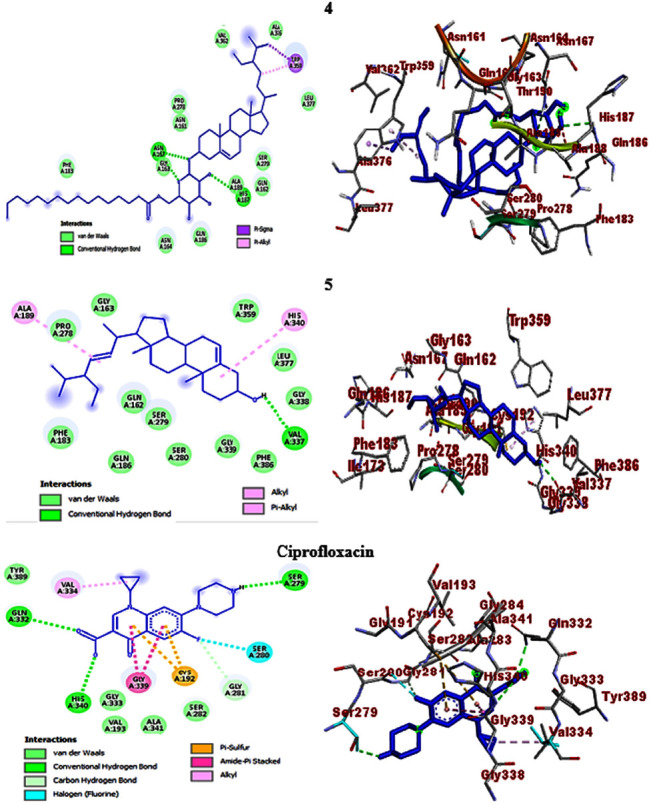
2D (left) and 3D (right) binding interactions of compounds **4**, **5**, and ciprofloxacin against *S. pyogenes* 10782 streptopain.

The molecular docking studies also revealed H-bond and residual amino acid interactions with the selected protein pockets. Accordingly, the binding affinity of *β*-sitosteryl-3*β*-glucopyranoside-6′-*O*-palmitate (**4**) with DNA gyrase B was stabilized by H-bond interactions with Arg-76, hydrophobic/electrostatic interactions with Ala-47, Ala-53, Ile-78, Arg-76, Pro-79, Val-43, Val-71, and Val-167 and Van der Waals interactions with Ile-94, Glu-50, Asn-46, Gly-77, Met-166, Thr-165, His-55, Asp-73, and Asp-49. H-bond interactions with Gly-77, Thr-165, and Asp-73 also stabilize the binding interactions of stigmasterol (**5**). Against *P. aeruginosa* PqsA, *β*-sitosteryl-3*β*-glucopyranoside-6′-*O*-palmitate (**4**) revealed H-bond interactions with Ala-245 and Gln-28 (C-H bond) and stigmasterol (**5**) with only Pro-326. In their binding interactions with *S. aureus* PK, *β*-sitosteryl-3*β*-glucopyranoside-6′-*O*-palmitate (**4**) showed three H-bonds with Lys-342, Lys-349, and Ser-345, while stigmasterol (**5**) has only one with Gln-338 (C-H bond). Furthermore, the computed binding affinities of the two compounds against *S. pyogenes* 10782 streptopain were mediated by H-bond interactions with Asn-167 and His-187 (*β*-sitosteryl-3*β*-glucopyranoside-6′-*O*-palmitate, **4**) and with only Val-337 (stigmasterol, **5**). Overall, the *in silico* molecular docking studies agreed with the *in vitro* antibacterial activities of the isolated compounds (**4** and **5**), and both compounds displayed comparable docking scores with the ciprofloxacin standard. All the docking information including binding affinities, H-bond, and residual amino acid interactions of the compounds and ciprofloxacin are summarized in Table 5, and their 2D and 3D interactions are depicted in Figures 5, 6, 7, and 8.

**Figure 9:**
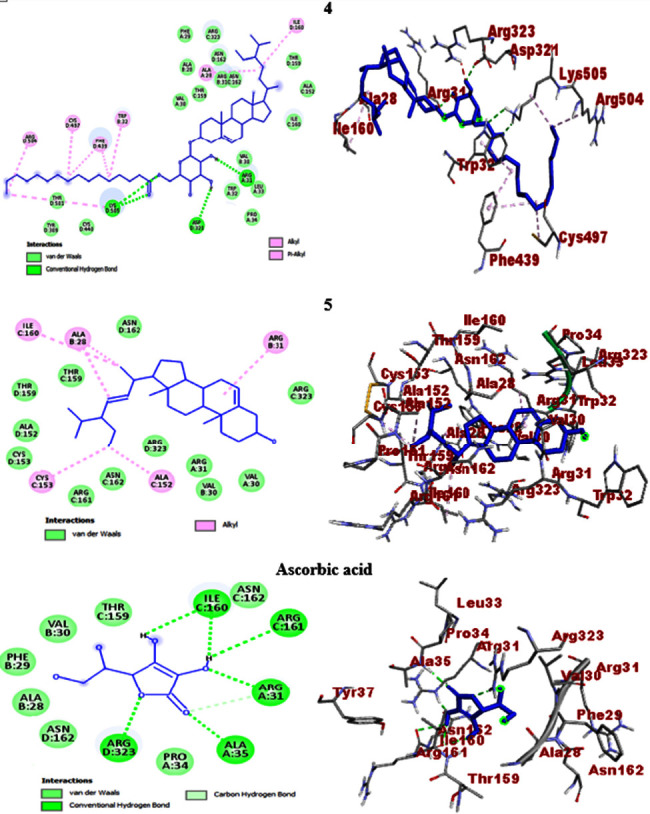
2D (left) and 3D (right) binding interactions of compounds **4**, **5**, and ascorbic acid against human myeloperoxidase.

The molecular docking study of *β*-sitosteryl-3*β*-glucopyranoside-6′-*O*-palmitate (**4**) and stigmasterol (**5**) was extended against human myeloperoxidase and the results revealed smooth relationships with the *in vitro* experimental data. Thereby, *β*-sitosteryl-3*β*-glucopyranoside-6′-*O*-palmitate (**4**) exhibited better binding affinity (−7.9 kcal/mol) than stigmasterol (**5**) (−7.7 kcal/mol). The scores were closer to the ascorbic acid standard (−8.0 kcal/mol), hence good scavenging activities. The greater binding affinity of *β*-sitosteryl-3*β*-glucopyranoside-6′-*O*-palmitate (**4**) can be associated with many hydroxyl groups in the glucose moiety, forming more than one H-bond interaction with the amino acids. Accordingly, *β*-sitosteryl-3*β*-glucopyranoside-6′-*O*-palmitate (**4**) formed three H-bond interactions with Lys-505, Arg-31, and Asp-321 and no H-bond interaction was observed by stigmasterol (**5**). The H-bonds, hydrophobic/electrostatic, and Van der Waals amino acid interactions of the two compounds along with ascorbic acid are summarized in Table 6 and their 2D and 3D orientations are portrayed in Figure 9.

## 4. Conclusion

In the present study, five compounds were isolated from the combined CH_2_Cl_2_ : CH_3_OH (1 : 1) and CH_3_OH root extracts of *Z. spina-christi*, and their *in vitro* antioxidant and antibacterial activities along with *in silico* computations were performed. The crude extract and compound isolates exhibited substantial activities against the bacterial strains. In addition, the DPPH radical scavenging activities revealed auspicious scavenging potential for CH_2_Cl_2_ : CH_3_OH (1 : 1) extract (IC_50_ : 1.51 *µ*g/mL) and *β*-sitosteryl-3*β*-glucopyranoside-6′-*O*-palmitate (**4**) (IC_50_ : 5.41 *µ*g/mL) compared to ascorbic acid (IC_50_ : 0.46 *µ*g/mL). The organ toxicity predictions of the compounds revealed that stearic acid (**2**) and 13-hydroxyoctadeca-9, 11-dienoic acid (**3**) were inactive towards all types of toxicities. The molecular docking findings in combination with the *in vitro* experimental data offer prospects for the advancement of novel therapeutic agents to combat various human ailments. Thus, the *in vitro* antioxidant and antibacterial studies together with the computational analyses suggest the potential use of the extract and stigmasterol (**5**) as antibacterial and antioxidant agents, and we recommend further investigations on other biological activities of *Z. spina-christi* and the compound isolates to consolidate its traditional welfares.

## Figures and Tables

**Table 1:** Zones of inhibition (mean ± SD) of CH_2_Cl_2_ : CH_3_OH (1 : 1) extract and the compound isolates (**1**–**5**).

Bacterial strain	Conc. (mg/mL)	Zone of inhibition (mm)						
		CH_2_Cl_2_ : CH_3_OH (1 : 1) extract	1	2	3	4	5	Ciprofloxacin (0.25 mg/mL)
*E. coli*	2	15.25 ± 0.35	12.50 ± 0.70	11.00 ± 0.00	12.25 ± 0.35	14.25 ± 0.35	13.50 ± 0.00	21.00 ± 0.35
	1	13.00 ± 0.00	10.00 ± 0.00	9.00 ± 0.00	9.50 ± 0.70	12.00 ± 0.00	11.25 ± 0.35	
	0.5	11.00 ± 0.00	8.00 ± 0.70	7.50 ± 0.35	8.00 ± 0.00	9.25 ± 0.35	8.50 ± 0.70	

*S. aureus*	2	14.25 ± 0.35	12.00 ± 0.00	11.00 ± 0.00	11.50 ± 0.70	12.25 ± 0.35	12.50 ± 0.35	21.50 ± 0.70
	1	12.00 ± 0.00	9.75 ± 0.35	8.50 ± 0.00	9.25 ± 0.35	10.50 ± 0.70	10.75 ± 0.35	
	0.5	9.75 ± 0.35	8.00 ± 0.00	NA	8.00 ± 0.00	8.50 ± 0.70	9.00 ± 0.70	

*P. aeruginosa*	2	14.50 ± 0.70	12.75 ± 0.35	11.00 ± 0.70	11.50 ± 0.00	13.50 ± 0.00	13.00 ± 0.00	22.50 ± 0.35
	1	11.75 ± 0.35	9.75 ± 0.35	9.00 ± 0.00	9.00 ± 0.70	11.25 ± 0.35	10.50 ± 0.35	
	0.5	9.50 ± 0.70	8.00 ± 0.00	7.00 ± 0.00	8.00 ± 0.70	9.25 ± 0.35	8.50 ± 0.70	

*S. pyogenes*	2	14.00 ± 0.00	12.25 ± 0.35	11.50 ± 0.00	11.75 ± 0.35	12.75 ± 0.35	13.50 ± 0.70	21.00 ± 0.70
	1	11.50 ± 0.70	9.50 ± 0.35	8.00 ± 0.70	8.75 ± 0.35	9.75 ± 0.70	9.75 ± 0.35	
	0.5	10.00 ± 0.00	7.00 ± 0.00	NA	NA	7.50 ± 0.70	7.50 ± 0.70	

NA; no activity, SD; standard deviation. For CH_2_Cl_2_ : CH_3_OH (1 : 1) extract, 2; 50 mg/mL, 1; 25 mg/mL, and 0.5; 12.5 mg/mL.

**Table 2:** Radical (DPPH) scavenging activity (%) of the CH_2_Cl_2_ : CH_3_OH (1 : 1) extract and compound isolates (**1**–**5**) of *Z. spina-christi.*

Conc. (*µ*g/mL)	Scavenging activity (%, mean ± SD)						
	CH_2_Cl_2_ : MeOH (1 : 1) extract	1	2	3	4	5	Ascorbic acid
62.5	73.93 ± 0.32	63.88 ± 0.23	57.87 ± 0.08	60.54 ± 0.00	64.55 ± 0.22	61.94 ± 0.00	79.62 ± 0.11
125	78.73 ± 0.20	68.95 ± 0.18	63.03 ± 0.22	64.76 ± 0.12	69.02 ± 0.27	65.74 ± 0.20	86.38 ± 0.13
250	82.96 ± 0.16	73.64 ± 0.35	67.21 ± 0.32	68.86 ± 0.30	73.95 ± 0.00	69.32 ± 0.23	89.05 ± 0.05
500	87.81 ± 0.14	76.33 ± 0.05	71.88 ± 0.02	73.26 ± 0.05	78.28 ± 0.00	73.50 ± 0.13	91.99 ± 0.03
1000	91.81 ± 0.28	83.29 ± 0.22	75.80 ± 0.06	77.00 ± 0.03	81.12 ± 0.15	76.86 ± 0.06	98.30 ± 0.00
IC_50_	1.51	7.67	17.46	10.59	5.41	6.88	0.46

**Table 3:** ADME and drug-likeness properties of the compound isolates (**1**–**5**) predicted by the SwissADME.

Predicted parameters	Compounds					
	1	2	3	4	5	Ciprofloxacin
*Drug likeness*						
MF	C_60_H_104_O_6_	C_18_H_36_O_2_	C_18_H_32_O_3_	C_51_H_90_O_7_	C_29_H_48_O	C_17_H_18_FN_3_O_3_
MW (g/mol)	921.46	284.48	296.44	815.26	412.69	331.34
NRB	50	16	14	25	5	3
NHD	0	1	2	3	1	2
NHA	6	2	3	7	1	5
LogP (iLogP)	12.02	4.30	3.72	8.43	5.01	2.24
TPSA (Å^2^)	78.90	37.30	57.53	105.45	20.23	74.57
Violations (LRo5)	2	1	0	2	1	0
Veber's rule		No	No	No	Yes	Yes

*ADME predictions*						
LogKp (cm/s)	3.85	−2.19	−4.31	−0.56	−2.74	−9.09
GIA	Low	High	High	Low	Low	High
BBB	No	No	Yes	No	No	No

*Inhibitor interaction*						
P-gp substrate	Yes	No	No	Yes	No	Yes
CYP1A2	No	Yes	Yes	No	No	No
CYP2C19	No	No	No	No	No	No
CYP2C9	No	No	Yes	No	Yes	No
CYP2D6	No	No	Yes	No	No	No
CYP3A4	No	No	No	Yes	No	No

MF; molecular formula, MW; molecular weight, NRB; number of rotatable bonds, NHD; number of hydrogen donors, NHA; number of hydrogen acceptors, LogP; lipophilicity, TPSA; total polar surface area, LRo5; Lipinski's rule of 5, LogKp; skin permeation value, GIA; gastrointestinal absorption, BBB; blood-brain barrier, P-gp; p glycoprotein, and CYP; cytochrome-P.

**Table 4:** Toxicity analysis of the compound isolates (**1**–**5**) predicted by ProTox-II online web tool.

Compound no	LD_50_ (mg/kg)	Toxicity class	Toxicity				
			Immuno	Mutagen	Cyto	Hepato	Carcino
1	37000	6	No	No	No	No	Yes
2	900	4	No	No	No	No	No
3	3200	5	No	No	No	No	No
4	4000	5	Yes	No	No	No	No
5	890	4	Yes	No	No	No	No
Ciprofloxacin	2000	4	No	Yes	No	No	No

LD; lethal dose, Immuno; immunotoxicity, Mutagen; mutagenicity, Cyto; cytotoxicity, Hepato; hepatotoxicity, and Carcino; carcinogenicity.

**Table 5:** Molecular docking profile of the compound isolates (**4** and **5**) and ciprofloxacin standard against *E. coli* DNA gyrase B, *P. aeruginosa* PqsA, *S. aureus* PK, and *S. pyogenes* 10782 streptopain.

Compound/ligand	Binding affinity (kcal/mol)	H-bond	Residual amino acid interactions			
			Hydrophobic	Electrostatic	Halogen	Van der Waals
*Against E. coli DNA gyrase B*						
**4**	−7.3	Arg-76	Ala-47, Arg-76, Ala-53, Ile-78, Val-43, Pro-79, Val-71, and Val-167	—	—	Ile-94, Asn-46, Glu-50, Gly-77, Met-166, Thr-165, Asp-73, His-55, and Asp-49
**5**	−7.0	Gly-77, Asp-73, and Thr-165	Ile-78 and Ile-94	—	—	Asn-46, Ala-47, Gly-164, Gly-75, Glu-50, Arg-76, Pro-79, Val-93, and Val-97
Ciprofloxacin	−7.6	Asn-46, Asp-73, and Arg-76	Asn-46, Ile-94, and Ile-78	Glu-50	Glu-50, Arg-76, and Gly-77	Pro-79, Thr-165, and Ala-47

*Against P. aeruginosa PqsA*						
**4**	−8.0	Ala-245 and Gln-28	Arg-247, Ala-21, and Leu-33	—	—	Gly-198, Thr-20, His-24, Leu-228, Phe-246, Gly-27, Asp-19, Ser-270, Gln-34, Ser-31, Leu-30, Ala-245, Gln-28, Thr-29, Arg-200, Ala-21, His-24, and Ser-226
**5**	−7.8	Pro-326	Pro-328, Pro-326, Leu-325, and Leu-182	—	—	Gly-183, Glu-343, Pro-356, Cys-332, Glu-331, Gly-329, Tyr-330, Arg-314, Glu-191, Arg-386, Gly-390, and Arg-186
Ciprofloxacin	−8.2	Ala-21, Arg-200, Thr-29, Asp-19, and Gln-34	His-24 and Ala-21	—	Asp-19 and Thr-20	Gly-27, Ser-31, Ala-245, Arg-247, Phe-246, Arg-200, Thr-29, and His-24

*Against S. aureus PK*						
**4**	−7.6	Lys-342, Lys-349, and Ser-345	Lys-260, Lys-342, and Leu-343	—	—	Asp-303, Ser-345, Tyr-302, Asp-346, Lys-260, Asp-339, Gln-338, Leu-343, Ala-337, and Arg-264
**5**	−7.7	Gln-338	Lys-260, Tyr-302, and Arg-264	—	—	Asp-261, Asn-299, Asp-303, Asp-339, and Ala-337
Ciprofloxacin	−7.9	Thr-366, Ser-362, Asn-369, and Ala-358	Ile-361 and His-365	—	—	Thr-353, Asn-369, Thr-348, His-365, Ile-361, and Ser-362

*Against S. pyogenes 10782 streptopain*						
**4**	−5.9	Asn-167 and His-187	Trp-359	—	—	Phe-183, Gly-163, Asn-161, Pro-278, Val-362, Ala-376, Leu-377, Ser-279, Gln-162, Ala-189, Gln-186, and Asn-164
**5**	−7.4	Val-337	Ala-189 and His-340	—	—	Pro-278, Gly-163, Trp-359, Leu-377, Gly-338, Phe-386, Gly-339, Ser-280, Ser-279, Gln-162, Gln-186, and Phe-183
Ciprofloxacin	−7.6	Gln-332, Ser-279, His-340, and Gly-281	Gly-339 and Val-334	Cys-192	Ser-280	Tyr-389, Ser-282, Ala-341, Gly-333, and Val-193

**Table 6:** Molecular docking profile of the compound isolates (**4** and **5**) and ascorbic acid standard against human myeloperoxidase.

Compound/ligand	Binding affinity (kcal/mol)	H-bond	Residual amino acid interactions	
			Hydrophobic	Van der Waals
**4**	−7.9	Lys-505, Arg-31, and Asp-321	Ala-28, Ile-160, Cys-497, Arg-504, Lys-505, Trp-32, and Phe-439	Val-30, Thr-159, Ala-28, Arg-31, Asn-162, Phe-29, Arg-323, Ala-152, Ile-160, Leu-33, Trp-32, Pro-34, Cys-440, Tyr-309, and Thr-501
**5**	−7.7	—	Ala-152, Ala-28, Ile-160, Arg-31, and Cys-153	Cys-153, Ala-152, Thr-159, Asn-162, Val-30, Arg-323, Arg-31, and Arg-161
Ascorbic acid	−8.0	Arg-31, Ala-35, Arg-323, Ile-160, and Arg-161	—	Ala-28, Phe-29, Val-30, Thr-159, Asn-162, and Pro-34

## Data Availability

The IR and NMR spectra used to support the present study are depicted in the Supplementary Materials. Further data can also be obtained from the corresponding author upon reasonable request.

## Supplementary Materials

Supplementary Materials The IR and NMR data of the isolated compounds are portrayed in the Supplementary Materials (Tables S1–7 and Figures S1–20).
