# Supplementary material for: In Vitro Antibacterial and Antioxidant Activities, Pharmacokinetics, and In Silico Molecular Docking Study of Phytochemicals from the Roots of Ziziphus spina-christi
Source: Biochem Res Int. 2024 Aug 9;2024:7551813. doi: 10.1155/2024/7551813 (PMC11390196; doi:10.1155/2024/7551813)
Supplement: Supplementary Materials — The IR and NMR data of the isolated compounds are portrayed in the Supplementary Materials (Tables S1–7 and Figures S1–20). [file 7551813.f1.pdf]

Supplementary Materials

In Vitro Antibacterial and Antioxidant Activities, Pharmacokinetics, and *In Silico* Molecular Docking Study of Phytochemicals from the Roots of *Ziziphus spina-christi*

Hadush Gebrehiwot[1], Urgessa Ensermu[2], Aman Dekebo[1, 3], Milkyas Endale[4], and Tariku Nefo Duke[5]

[1]*Department of Applied Chemistry, Adama Science and Technology University, P.O. Box 1888, Adama, Ethiopia*
[2]*Department of Applied Biology, Adama Science and Technology University, P.O. Box 1888, Adama, Ethiopia*
[3]*Institute of Pharmaceutical Sciences, Adama Science and Technology University, P.O. Box 1888, Adama, Ethiopia*
[4]*Traditional and Modern Medicine Research and Development Directorate, Armauer Hansen Research Institute, P.O. Box 1242, Addis Ababa, Ethiopia*
[5]*Department of Materials Science and Engineering, National Taiwan University of Science and Technology, No.43, Keelung Rd., Sec.4, Taipei 10607, Taiwan*

Correspondence should be addressed to Milkyas Endale; milkyasendale@yahoo.com

In this file, the tabular and spectral IR and NMR information used to establish the chemical structures of the isolated compounds from the roots of *Ziziphus spina-christi* are depicted in Tables S1-7 and Figures S1-20, respectively.

TABLE S1: FTIR absorption frequencies of compound **1**.

**Observed frequency (cm^{-1})**	**Possible frequency range (cm^{-1})**	**Assignments**
2918	2850-3000	Asymmetrical -C-H stretching in alkanes
2849	2850-2950	Symmetrical -C-H stretching in alkanes
1739	1650-1850	-C=O stretching of esters
1455	1450-1475	$-CH_2$ scissoring vibration in aliphatic region
1373	1350-1470	-C-H bending vibration in alkanes
1162	1150-1280	-C-O-C stretching in esters
973	950-1000	=CH out plane deformation in olefines
721	665-1000	-O-C=O bending vibration in esters

TABLE S2: ^{1}H (600 MHz), ^{13}C, and DEPT-135 NMR ($CDCl_3$, δ in ppm, J in Hz) spectral data of compound **1** and ^{1}H and ^{13}C NMR values of trimethyl trilinolein in the literature.

**C. No.**	**Compound 1**			**trimethyl trilinolein** [54, 55]	
	**^{1}H NMR**	**^{13}C NMR**	**DEPT-135**	**^{1}H NMR**	**^{13}C NMR**
1a, 1b	4.17 (1H, *dd*, J = 11.8, 5.8), 4.32 (1H, *dd*, J = 12.0, 4.4)	62.1	$-CH_2-O-$	4.12 (1H, *dd*, J = 14.0, 6.0), 4.28 (1H, *dd*, J = 14.0, 4.2)	62.1
2	5.29 (1H, *m*)	68.9	-CH-O-	5.25 (1H, *m*)	68.9
3a, 3b	4.17 (1H, *dd*, J = 11.8, 5.8), 4.32 (1H, *dd*, J = 12.0, 4.4)	62.1	$-CH_2-O-$	4.12 (1H, *dd*, J = 14.0, 6.0), 4.28 (1H, *dd*, J = 14.0, 4.2)	62.1
1', 1''	-	173.2, 172.1	Q	-	173.3, 172.8
2', 2''	2.33 (6H, *t*, J = 7.6)	34.0, 34.2	$-CH_2-$	2.28 (4H, *t*, J = 7.6), 2.29 (2H, *t*, J = 7.6)	34.0, 34.2
3', 3''	1.63 (6H, *m*)	24.8, 25.6	$-CH_2-$	1.59 (6H, *m*)	24.8
4', 4''	1.28 (6H, *m*)	29.2, 29.1	$-CH_2-$	1.25 (6H, *m*)	29.1, 29.0
5', 5''	1.28 (6H, *m*)	29.4	$-CH_2-$	1.25 (6H, *m*)	29.2, 29.3
6', 6''	1.28 (6H, *m*)	29.3, 29.3	$-CH_2-$	1.25 (6H, *m*)	29.1, 29.2
7', 7''	1.28 (6H, *m*)	29.7	$-CH_2-$	1.25 (6H, *m*)	29.6
8', 8''	2.07 (6H, *m*)	32.2	$-CH_2-$	2.02 (6H, *m*)	27.2
9', 9''	5.38 (3H, *m*)	130.2	=CH-	5.33 (3H, *m*)	130.0
10', 10''	5.40 (3H, *m*)	127.9	=CH-	5.33 (3H, *m*)	128.0
11', 11''	2.79 (6H, *d*, J = 6.9)	39.7	$-CH_2-$	2.75 (4H, *m*), 2.02 (2H, *m*)	29.7
12', 12''	-	135.2	Q	5.33 (2H, *m*), 1.25 (2H, *m*)	127.9
13', 13''	5.14 (3H, *t*, J = 7.4)	125.0	=CH-	5.33 (2H, *m*), 1.25 (2H, *m*)	130.2
14', 14''	2.07 (6H, *m*)	27.2	$-CH_2-$	2.02 (4H, *m*), 1.25 (2H, *m*)	27.1
15', 15''	1.28 (6H, *m*)	29.6	$-CH_2-$	1.25 (6H, *m*)	29.3
16', 16''	1.28 (6H, *m*)	31.9	$-CH_2-$	1.25 (6H, *m*)	31.9
17', 17''	1.33 (6H, *m*)	22.7	$-CH_2-$	1.25 (6H, *m*)	22.7
18', 18''	0.90 (9H, *brt*, J = 6.7)	14.1	$-CH_3$	0.86 (6H, *brt*), 0.87 (3H, *brt*)	14.1
19', 19''	1.70 (9H, *s*)	23.4	$-CH_3$	-	-

✓ The spectrum also displayed signals of acetone impurity at δ_H 2.19 (6H, *s*) and δ_C 206.9, and 30.9, and were out of the spectral analyses.

TABLE S3: FTIR absorption frequencies of compound **2**.

**Observed frequency (cm^{-1})**	**Possible frequency range (cm^{-1})**	**Assignments**
2554-3500	2400-3500	Hydrogen bonded –OH stretching in carboxylic acids
2918	2850-3000	Asymmetrical -C-H stretching in alkanes
2842	2840-2950	Symmetrical -C-H stretching in alkanes
1705	1690-1710	-C=O stretching in carboxylic acids
1460	1450-1475	$-CH_2$ scissoring vibration in aliphatic compounds
1376	1350-1470	Asymmetrical $–CH_3$ deformation in alkanes
1167	1150-1280	-C-O stretching in carboxylic acids
722	665-1000	-O-C=O bending vibration in carboxylic acids

TABLE S4: ^{1}H (600 MHz), ^{13}C, and DEPT-135 NMR ($CDCl_3$, δ in ppm, J in Hz) spectral data of compound **2** and ^{1}H and ^{13}C NMR values of stearic acid in the literature.

**C No.**	**Compound 2**			**Stearic acid** [56]	
	**^{1}H NMR**	**^{13}C NMR**	**DEPT-135**	**^{1}H NMR**	**^{13}C NMR**
1	-	179.3	Q (C=O)	-	178.7
2	2.37 (2H, *t*, J = 7.6)	33.9	$-CH_2-$	2.34 (2H, *t*, J = 7.5)	33.8
3	1.66 (2H, *m*)	24.6	$-CH_2-$	1.63 (2H, *m*)	24.7
4	1.28-1.32 (2H, *m*)	29.0	$-CH_2-$	1.28-1.30 (2H, *m*)	29.7-30.0
5	1.28-1.32 (2H, *m*)	29.2	$-CH_2-$	1.28-1.30 (2H, *m*)	29.7-30.0
6	1.28-1.32 (2H, *m*)	29.6	$-CH_2-$	1.28-1.30 (2H, *m*)	29.7-30.0
7	1.28-1.32 (2H, *m*)	29.6	$-CH_2-$	1.28-1.30 (2H, *m*)	29.7-30.0
8	1.28-1.32 (2H, *m*)	29.6	$-CH_2-$	1.28-1.30 (2H, *m*)	29.7-30.0
9	1.28-1.32 (2H, *m*)	29.6	$-CH_2-$	1.28-1.30 (2H, *m*)	29.7-30.0
10	1.28-1.32 (2H, *m*)	29.6	$-CH_2-$	1.28-1.30 (2H, *m*)	29.7-30.0
11	1.28-1.32 (2H, *m*)	29.6	$-CH_2-$	1.28-1.30 (2H, *m*)	29.7-30.0
12	1.28-1.32 (2H, *m*)	29.6	$-CH_2-$	1.28-1.30 (2H, *m*)	29.7-30.0
13	1.28-1.32 (2H, *m*)	29.6	$-CH_2-$	1.28-1.30 (2H, *m*)	29.7-30.0
14	1.28-1.32 (2H, *m*)	29.4	$-CH_2-$	1.28-1.30 (2H, *m*)	29.7-30.0
15	1.28-1.32 (2H, *m*)	29.3	$-CH_2-$	1.28-1.30 (2H, *m*)	29.7-30.0
16	1.28-1.32 (2H, *m*)	31.9	$-CH_2-$	1.28-1.30 (2H, *m*)	31.9
17	1.28-1.32 (2H, *m*)	22.7	$-CH_2-$	1.28-1.30 (2H, *m*)	22.7
18	0.90 (3H, *t*, J = 7.0)	14.1	$-CH_3$	0.88 (3H, *t*, J = 7.0)	14.1

TABLE S5: ^{1}H (600 MHz), ^{13}C, and DEPT-135 NMR ($CDCl_3$, δ in ppm, J in Hz) spectral data of compound **3** and ^{1}H and ^{13}C NMR values of 13-hydroxyoctadeca-9, 11-dienoic acid in the literature.

C No.	Compound 3			13-hydroxyoctadeca-9, 11-dienoic acid [57]	
	**^{1}H NMR**	**^{13}C NMR**	**DEPT-135**	**^{1}H NMR**	**^{13}C NMR**
1	-	179.7	Q (C=O)	-	179.5
2	2.37 (2H, *t*, *J* = 7.5)	33.7	-CH_2-	2.33 (2H, *t*, *J* =7.4)	34.1
3	1.60 (2H, *m*)	24.6	-CH_2-	1.62 (2H, *m*)	24.8
4	1.28-1.34 (2H, *m*)	29.1	-CH_2-	1.24-1.34 (2H, *m*)	29.0
5	1.28-1.34 (2H, *m*)	29.3	-CH_2-	1.24-1.34 (2H, *m*)	29.0
6	1.28-1.34 (2H, *m*)	29.7	-CH_2-	1.24-1.34 (2H, *m*)	29.0
7	1.28-1.34 (2H, *m*)	31.4	-CH_2-	1.34-1.42 (2H, *m*)	29.5
8	2.20 (2H, *m*)	27.7	-CH_2-	2.17 (2H, *q*, *J* = 9.8, 7.4, 4.1, 2.4)	27.7
9	5.48 (1H, *m*)	133.1	=CH-	5.43 (1H, *dt*, *J* = 10.8, 7.6)	132.9
10	5.99 (1H, *t*, *J* = 11.0)	127.6	=CH-	5.97 (1H, *t*, *J* = 11.0, 1.1)	128.0
11	6.51 (1H, *dd*, *J* = 15.2, 11.1)	125.9	=CH-	6.48 (1H, *m*, *J* = 15.2, 11.1, 1.2)	126.0
12	5.68 (1H, *dd*, *J* =15.2, 6.9)	135.6	=CH-	5.66 (1H, *dd*, *J* = 15.2, 6.8)	135.8
13	4.18 (1H, *q*, *J* = 6.6)	72.9	-CH-O-	4.17 (1H, *q*, *J* = 6.1)	73.1
14	1.52 (2H, *m*)	37.2	-CH_2-	1.53 (2H, *m*)	37.4
15	1.28-1.34 (2H, *m*)	25.3	-CH_2-	1.24-1.34 (2H, *m*)	25.2
16	1.28-1.34 (2H, *m*)	31.9	-CH_2-	1.24-1.34 (2H, *m*)	31.9
17	1.28-1.34 (2H, *m*)	22.7	-CH_2-	1.24-1.34 (2H, *m*)	22.7
18	0.91 (3H, *t*, *J* =7.3)	14.1	-CH_3	0.84-0.92 (3H, *t*, *J* =7.4)	14.2
OH	3.34 (1H, *s*)	-	-	-	-

- ✓ Signals at δ_H 2.19 (6H, s) and δ_C 207.4, and 30.9 attributed to some undried acetone impurity in the sample.

TABLE S6: ^{1}H (600 MHz), ^{13}C, DEPT-135, and COSY NMR ($CDCl_3$, δ in ppm, *J* in Hz) spectral data of compound **4** and ^{1}H and ^{13}C NMR values of *β*-sitosteryl-3*β*-glucopyranoside-6'-*O*-palmitate in the literature.

**C. No.**	**Compound 4**				***β*-sitosteryl-3*β*-glucopyranoside-6'-*O*-palmitate** [58]	
	**^{1}H NMR**	**^{13}C NMR**	**DEPT-135**	**COSY**	**^{1}H NMR**	**^{13}C NMR**
1	1.82 (1H, *m*), 1.06 (1H, *m*)	37.3	-CH_2-		1.85 (1H, *m*), 1.06 (1H, *m*)	37.3
2	1.91 (1H, *m*), 1.59 (1H, *m*)	29.8	-CH_2-		1.95 (1H, *m*), 1.61 (1H, *m*)	29.7
3	3.54 (1H, *m*)	79.7	-CH-O-		3.54 (1H, *m*)	79.6
4	2.26 (1H, *m*), 2.24 (1H, *m*)	38.9	-CH_2-		2.36 (1H, *m*), 2.27 (1H, *m*)	38.9
5	-	140.3	Q		-	140.3
6	5.34 (1H, *m*)	122.2	=CH-		5.38 (1H, *m*)	122.1
7	1.98 (1H, *m*), 1.92 (1H, *m*)	32.0	-CH_2-		1.98 (2H, *m*)	31.9
8	1.50 (1H, *m*)	31.9	-CH-		1.52 (1H, *m*)	31.9
9	0.91 (1H, *m*)	50.2	-CH-		0.93 (1H, *m*)	50.1
10	-	36.8	Q		-	36.7
11	1.54 (1H, *m*), 1.01 (1H, *m*)	21.2	-CH_2-		1.56 (1H, *m*), 1.02 (1H, *m*)	21.0
12	2.01 (1H, *m*), 1.14 (1H, *m*)	39.8	-CH_2-		2.02 (1H, *m*), 1.18 (1H, *m*)	39.7
13	-	42.4	Q		-	42.3
14	1.00 (1H, *m*)	56.8	-CH-		1.01 (1H, *m*)	56.7
15	1.13 (1H, *m*), 1.08 (1H, *m*)	24.3	-CH_2-		1.12 (1H, *m*), 1.08 (1H, *m*)	24.3
16	1.47 (1H, *m*), 1.45 (1H, *m*)	27.3	-CH_2-		1.86 (1H, *m*), 1.83 (1H, *m*)	28.2
17	1.12 (1H, *m*)	56.1	-CH-		1.12 (1H, *m*)	56.1
18	0.66 (3H, *s*)	11.9	-CH_3		0.68 (3H, *s*)	11.8
19	0.99 (3H, *s*)	19.4	-CH_3		1.00 (3H, *s*)	19.3
20	1.32-1.26 (1H, *m*)	36.2	-CH-		1.36 (1H, *m*)	36.2
21	0.87 (3H, *d*, *J* = 6.3)	18.8	-CH_3		0.92 (3H, *d*, *J* =6.4)	18.8
22	1.32-1.26 (2H, *m*)	33.8	-CH_2-		1.34 (1H, *m*), 1.00 (1H, *m*)	33.9
23	1.14 (2H, *m*)	25.5	-CH_2-		1.18 (2H, *m*)	26.1
24	0.91 (1H, *m*)	45.8	-CH-		0.95 (1H, *m*)	45.8
25	1.57 (1H, *m*)	29.2	-CH-		1.66 (1H, *m*)	29.1
26	0.80 (3H, *d*, *J* = 6.7)	19.1	-CH_3		0.82 (3H, *d*, *J* = 6.8)	19.0
27	0.81 (3H, *d*, *J* = 6.7)	19.9	-CH_3		0.84 (3H, *d*, *J* = 6.8)	19.8
28	1.24 (2H, *brs*)	23.1	-CH_2-		1.26 (2H, *m*)	23.0
29	0.82 (3H, *t*, *J* = 7.5)	12.3	-CH_3		0.84 (3H, *t*, *J* = 7.6)	12.0
	Glucopyranoside moiety					
1'	4.27 (1H, *d*, *J* = 11.5)	101.2	-CH-O-		4.38 (1H, *d*, *J* = 7.7)	101.2
2'	3.36 (1H, *m*)	73.6	-CH-O-		3.35 (1H, *dd*, *J* = 8.7, 7.7)	73.4
3'	3.65 (1H, *m*)	76.1	-CH-O-		3.57 (1H, *dd*, *J* = 9.9, 8.7)	76.0
4'	3.37 (1H, *m*)	70.1	-CH-O-		3.38 (1H, *dd*, *J* = 9.9, 8.6)	70.2
5'	3.44 (1H, *m*)	73.9	-CH-O-		3.45 (1H, *m*)	73.8

6'	4.37 (1H, *dd*, *J* = 12.1, 5.2), 4.13 (1H, *dd*, *J* = 12.1, 6.3)	63.4	-CH_2-O-		4.42 (1H, *dd*, *J* = 12.1, 5.3), 4.29 (1H, *dd*, *J* = 12.1, 1.7)	63.4
	Palmitoyl moiety					
1"	-	174.7	Q (-C=O)		-	174.6
2"	2.31 (2H, *t*, *J* = 7.5)	34.3	-CH_2-	H-2"↔H-3"	2.34 (2H, *t*, *J* = 7.6)	34.2
3"	1.61 (2H, *m*)	25.0	-CH_2-	H-3"↔H-2"	1.61 (2H, *m*)	24.9
4"	1.24 (2H, *brs*)	29.4	-CH_2-		1.28 (2H, *brs*)	29.2
5"	1.24 (2H, *brs*)	29.6	-CH_2-		1.26 (2H, *brs*)	29.4
6"	1.24 (2H, *brs*)	29.7	-CH_2-		1.26 (2H, *brs*)	29.6
7"-12"	1.24 (2H, *brs*)	29.8	-CH_2-		1.26 (2H, *brs*)	29.8
13"	1.24 (2H, *brs*)	29.4	-CH_2-		1.26 (2H, *brs*)	29.4
14"	1.24 (2H, *brs*)	31.9	-CH_2-		1.26 (2H, *brs*)	31.9
15"	1.24 (2H, *brs*)	22.8	-CH_2-		1.30 (2H, *brs*)	22.7
16"	0.86 (3H, *t*, *J* = 7.0)	14.2	-CH_3		0.88 (3H, *t*, *J* = 7.1)	14.1

Table S7: ^{1}H (600 MHz), ^{13}C, DEPT-135, and COSY NMR ($CDCl_3$, δ in ppm, J in Hz) spectral data of compound **5** and ^{1}H and ^{13}C NMR values of stigmasterol in the literature.

C No.	**Compound 5**				**Stigmasterol** [60]	
	**^{1}H NMR**	**^{13}C NMR**	**DEPT-135**	**COSY**	**^{1}H NMR**	**^{13}C NMR**
1	1.84 (1H, *m*), 1.09 (1H, *m*)	37.3	-CH_2-	H-1b↔H-2a	1.83 (1H, *m*), 1.08 (1H, *m*)	37.2
2	1.82 (1H, *m*), 1.50 (1H, *m*)	31.7	-CH_2-	H-2a↔H-1b	1.82 (1H, *m*), 1.49 (1H, *m*)	31.6
3	3.51 (1H, *m*)	71.9	-CH-O-	H-3↔H-4a	3.53 (1H, *m*)	71.8
4	2.25 (1H, *m*), 2.21 (1H, *m*)	42.4	-CH_2-	H-4a↔H-3	2.28 (1H, *m*), 2.24 (1H, *m*)	42.3
5	-	140.8	Q		-	140.7
6	5.35 (1H, *dd*, *J* = 5.0, 2.7)	121.8	=CH-	H-6↔H-7a	5.35 (1H, *d*, *J* = 4.7)	121.7
7	1.96 (1H, *m*), 1.51 (1H, *m*)	32.0	-CH_2-	H-7a↔H-6, 8	1.98 (1H, *m*), 1.53 (1H, *m*)	31.9
8	1.49 (1H, *m*)	31.9	-CH-	H-8↔H-7a	1.46 (1H, *m*)	31.9
9	0.92 (1H, *m*)	50.2	-CH-		0.94 (1H, *m*)	50.1
10	-	36.6	Q		-	36.5
11	1.47 (1H, *m*), 1.43 (1H, *m*)	21.1	-CH_2-		1.48 (1H, *m*), 1.45 (1H, *m*)	21.0
12	1.99 (1H, *m*), 1.16 (1H, *m*)	39.7	-CH_2-		1.97 (1H, *m*), 1.15 (1H, *m*)	39.7
13	-	42.3	Q		-	42.2
14	1.06 (1H, *m*)	56.9	-CH-		1.00 (1H, *m*)	56.8
15	1.52 (1H, *m*), 1.07 (1H, *m*)	24.4	-CH_2-	H-15↔H-16	1.55 (1H, *m*), 1.06 (1H, *m*)	24.4
16	1.81 (1H, *m*), 1.28 (1H, *m*)	29.8	-CH_2-	H-16↔H-15	1.71 (1H, *m*), 1.27 (1H, *m*)	28.9
17	1.14 (1H, *m*)	56.0	-CH-		1.13 (1H, *m*)	55.9
18	0.68 (3H, *s*)	12.1	-CH_3		0.70 (3H, *s*)	12.0
19	0.99 (3H, *s*)	19.5	-CH_3		1.01 (3H, *s*)	19.4
20	2.03 (1H, *m*)	40.6	-CH-	H-20↔H-21	2.04 (1H, *m*)	40.5
21	1.02 (3H, *d*, *J* = 7.2)	21.2	-CH_3	H-21↔H-20	1.02 (3H, *d*, *J* = 6.8)	21.1
22	5.14 (1H, *dd*, *J* = 15.1, 8.6)	138.4	=CH-	H-22↔H-23	5.15 (1H, *dd*, *J* = 15.1, 8.4)	138.3
23	5.00 (1H, *dd*, *J* = 15.2, 8.6)	129.3	=CH-	H-23↔H-22	5.02 (1H, *dd*, *J* = 15.1, 8.4)	129.2
24	1.54 (1H, *m*)	51.3	-CH-		1.53 (1H, *m*)	51.2
25	1.43 (1H, *m*)	31.9	-CH-	H-25↔H-26, 27	1.44 (1H, *m*)	31.9
26	0.82 (3H, *d*, *J* = 6.3)	21.3	-CH_3	H-26↔H-25	0.84 (3H, *d*, *J* = 6.4)	21.2
27	0.83 (3H, *d*, *J* = 6.1)	19.0	-CH_3	H-27↔H-25	0.83 (3H, *d*, *J* = 6.1)	18.9
28	0.90 (2H, *m*)	25.5	-CH_2-		1.15 (2H, *m*)	25.4
29	0.79 (3H, *t*, *J* = 6.0)	12.3	-CH_3		0.80 (3H, *t*, *J* = 6.0)	12.3

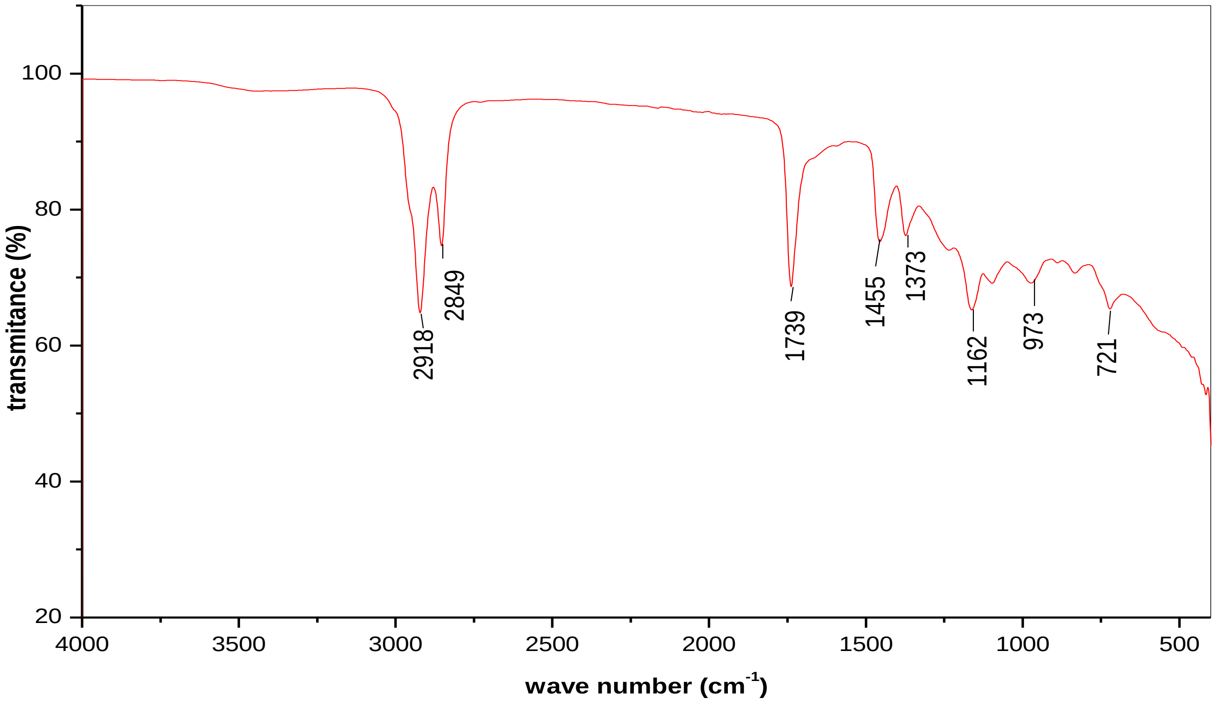

FIGURE S1: FTIR spectrum of compound **1**.

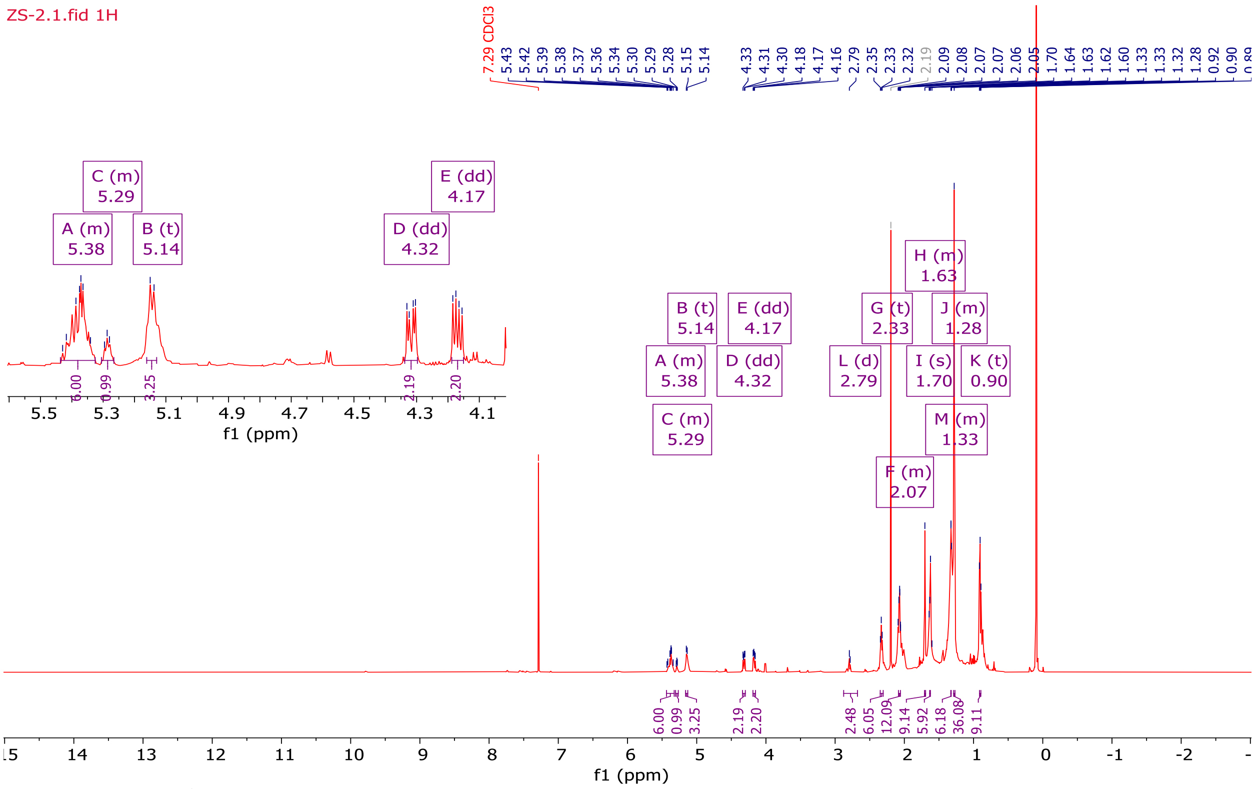

FIGURE S2: ^{1}H NMR spectrum of compound **1**.

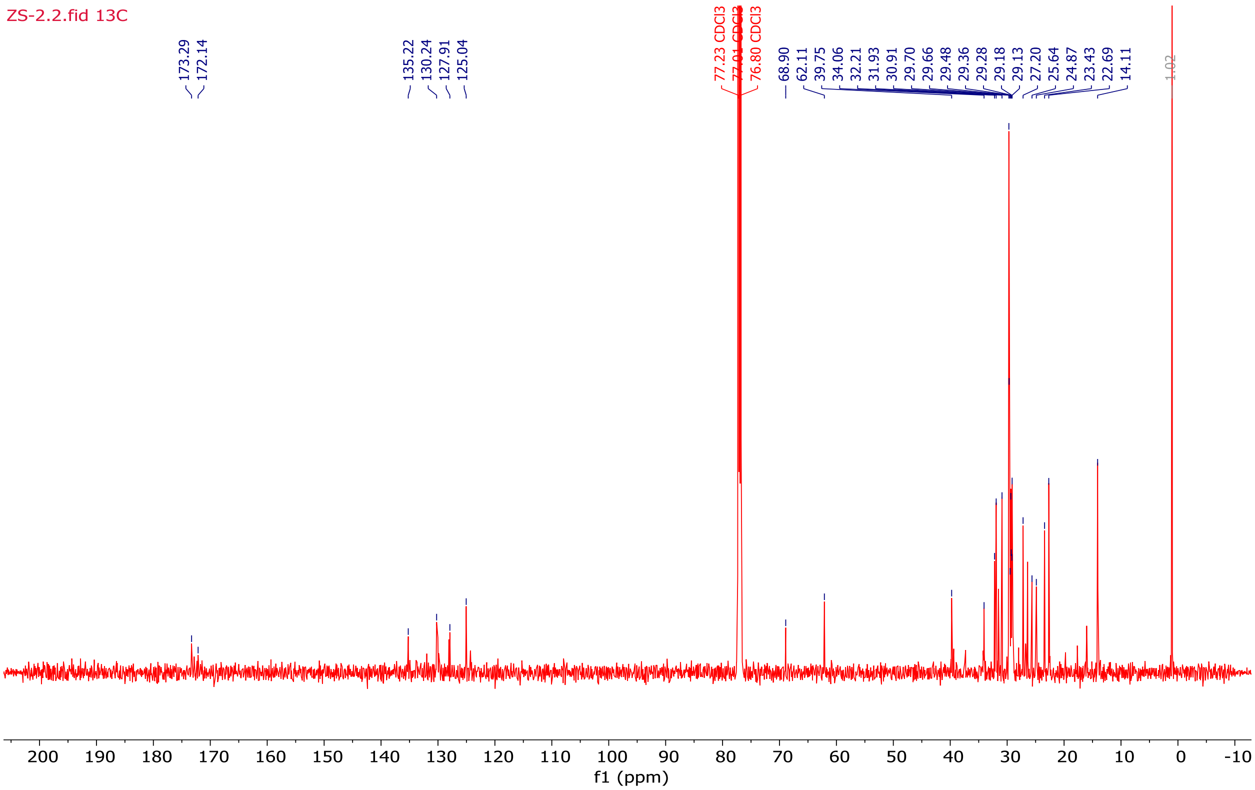

FIGURE S3: ^{13}C NMR spectrum of compound **1**.

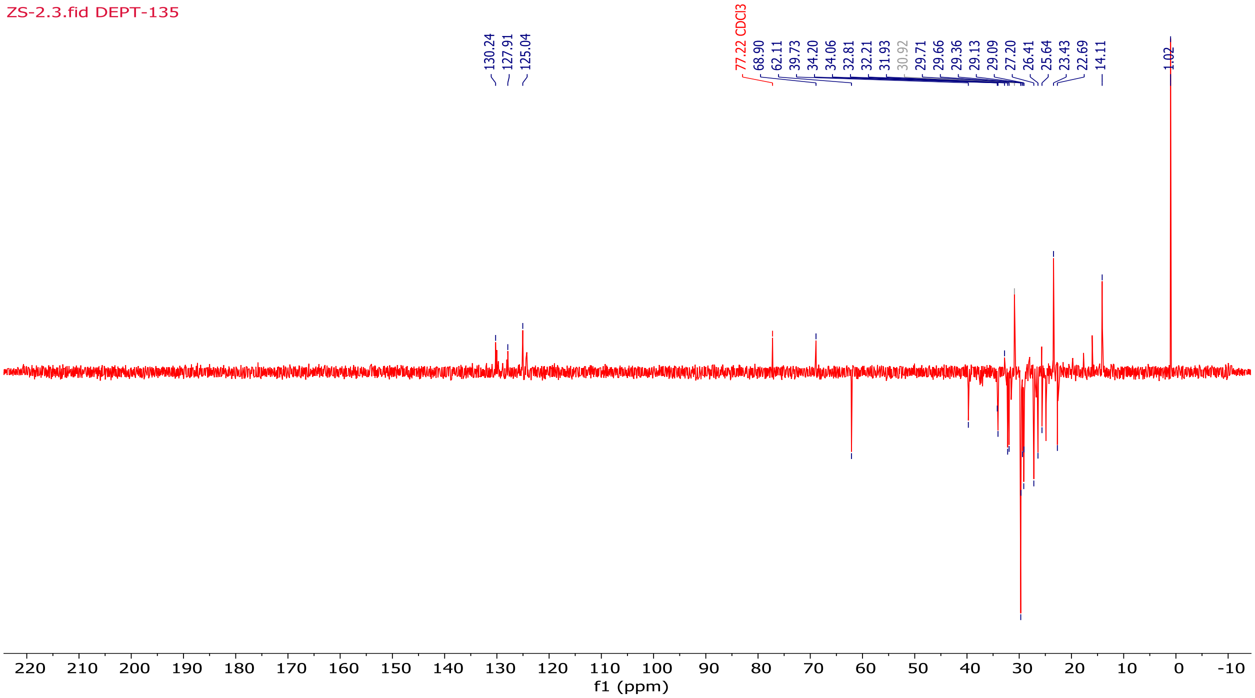

FIGURE S4: DEPT-135 spectrum of compound **1**.

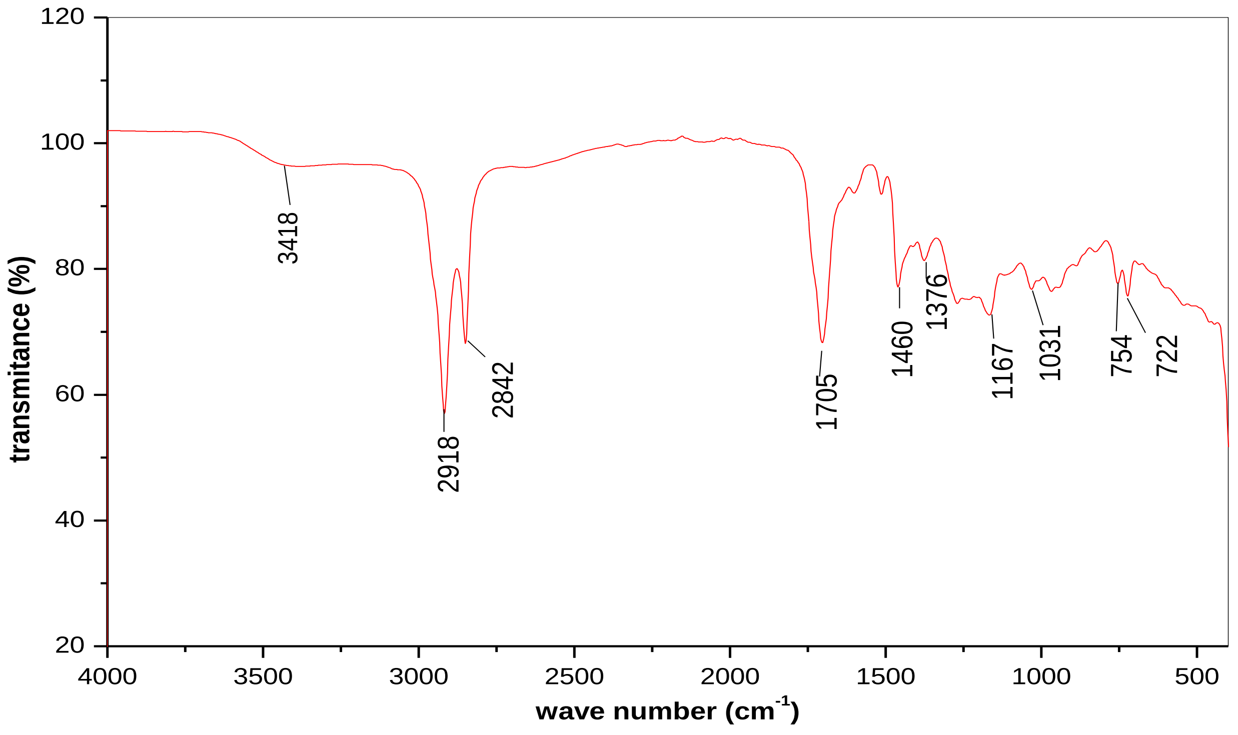

FIGURE S5: FTIR spectrum of compound **2**.

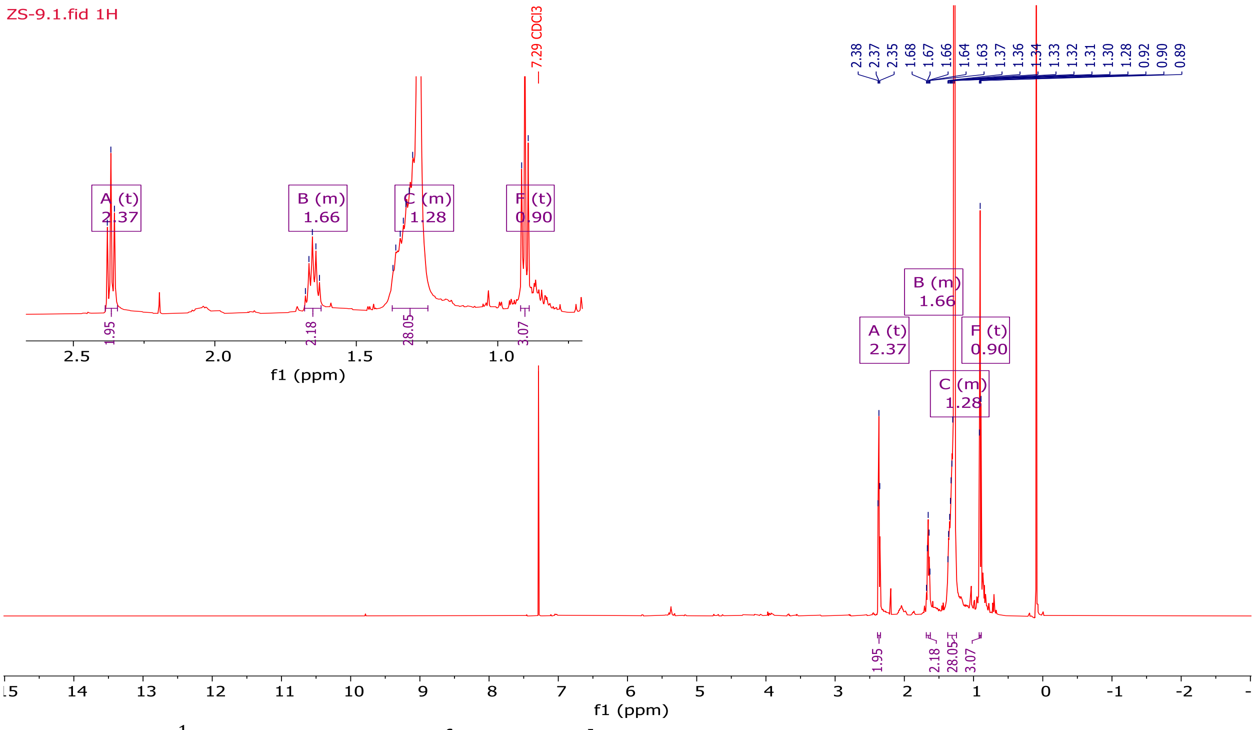

FIGURE S6: ^{1}H NMR spectrum of compound **2**.

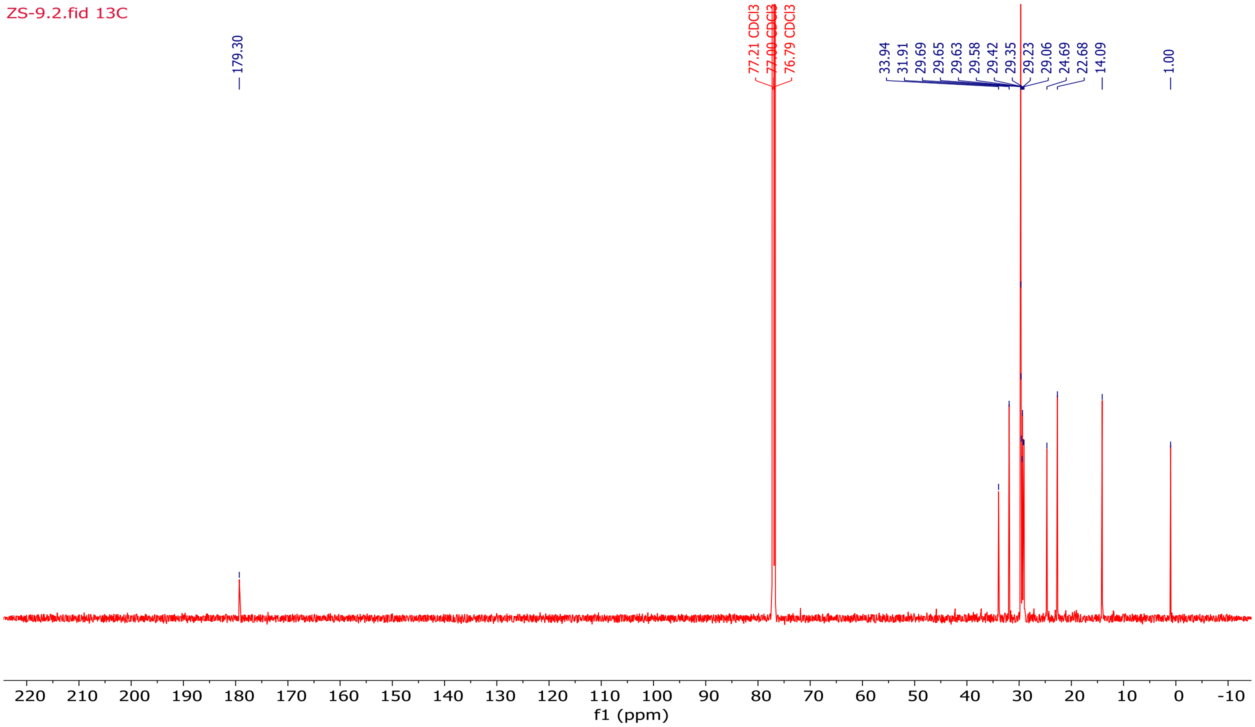

FIGURE S7: ^{13}C NMR spectrum of compound **2**.

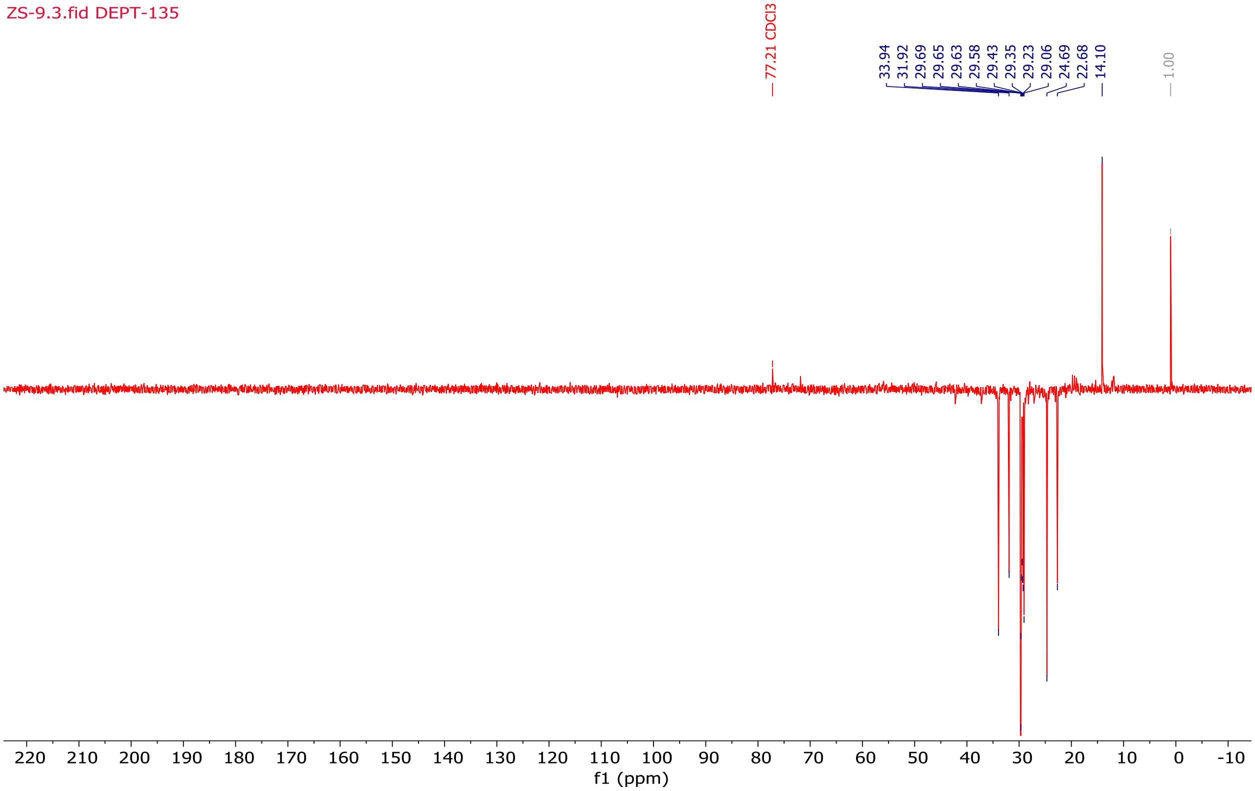

FIGURE S8: DEPT-135 spectrum of compound **2**.

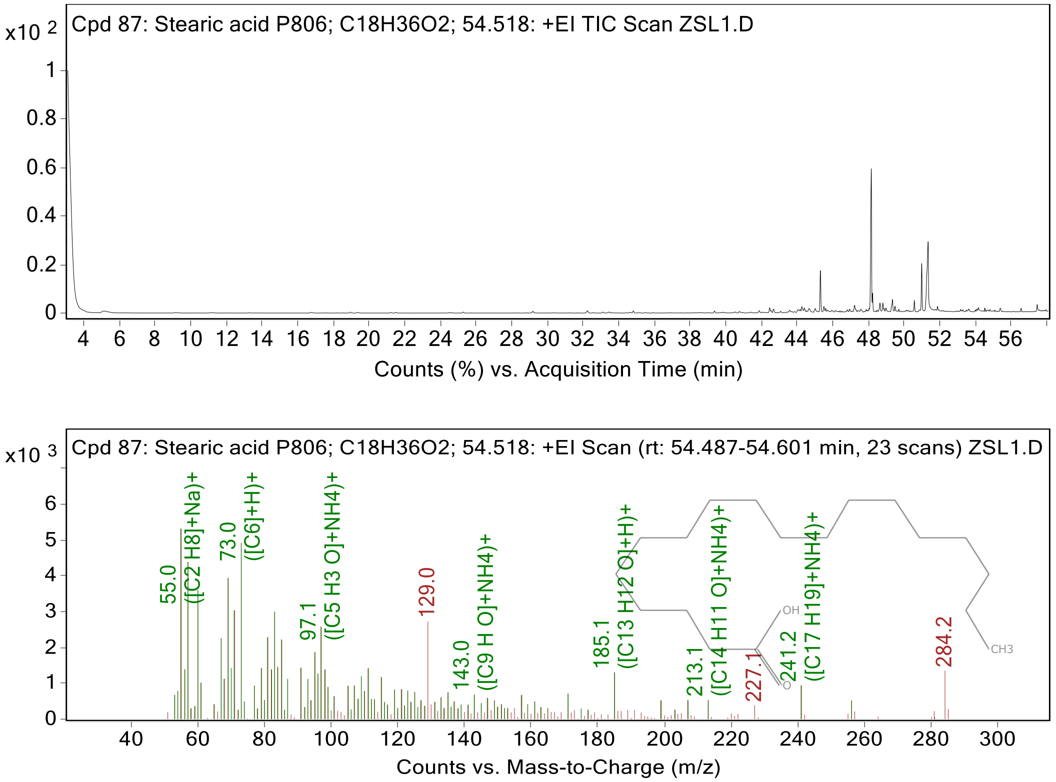

FIGURE S9: GC (top) and mass fragmentation pattern (bottom) of compound **2**.

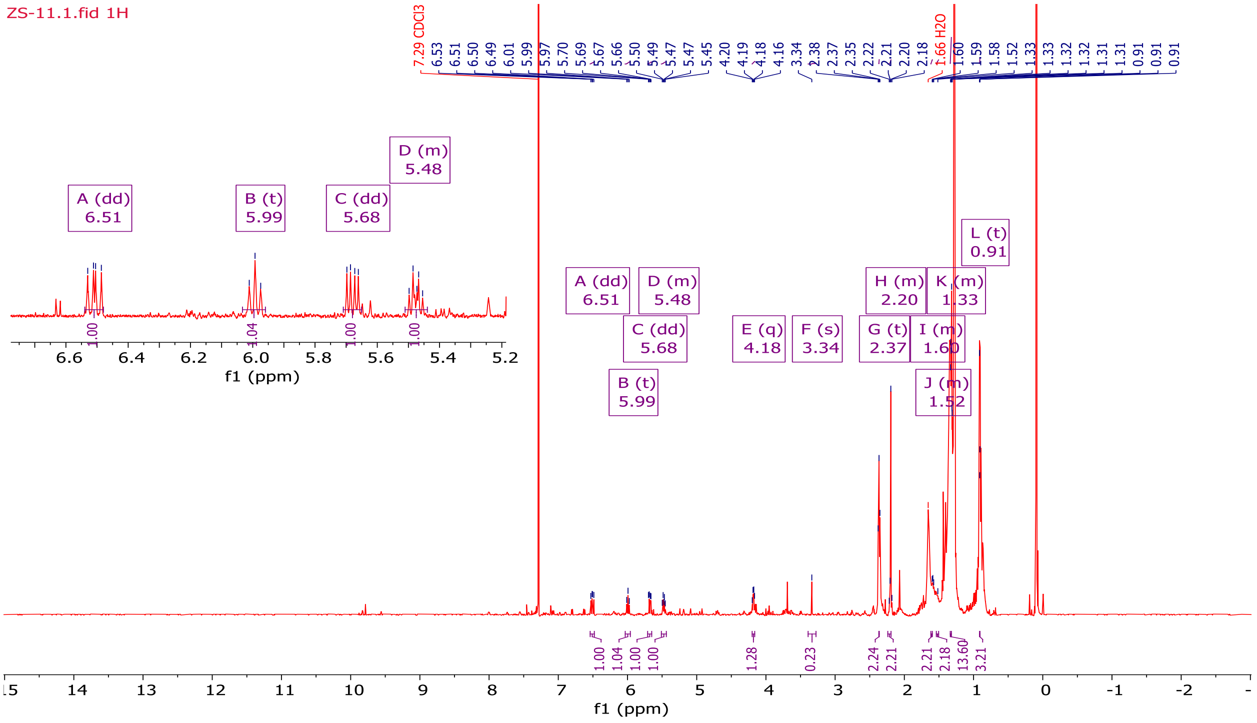

FIGURE S10: ^{1}H NMR spectrum of compound **3**.

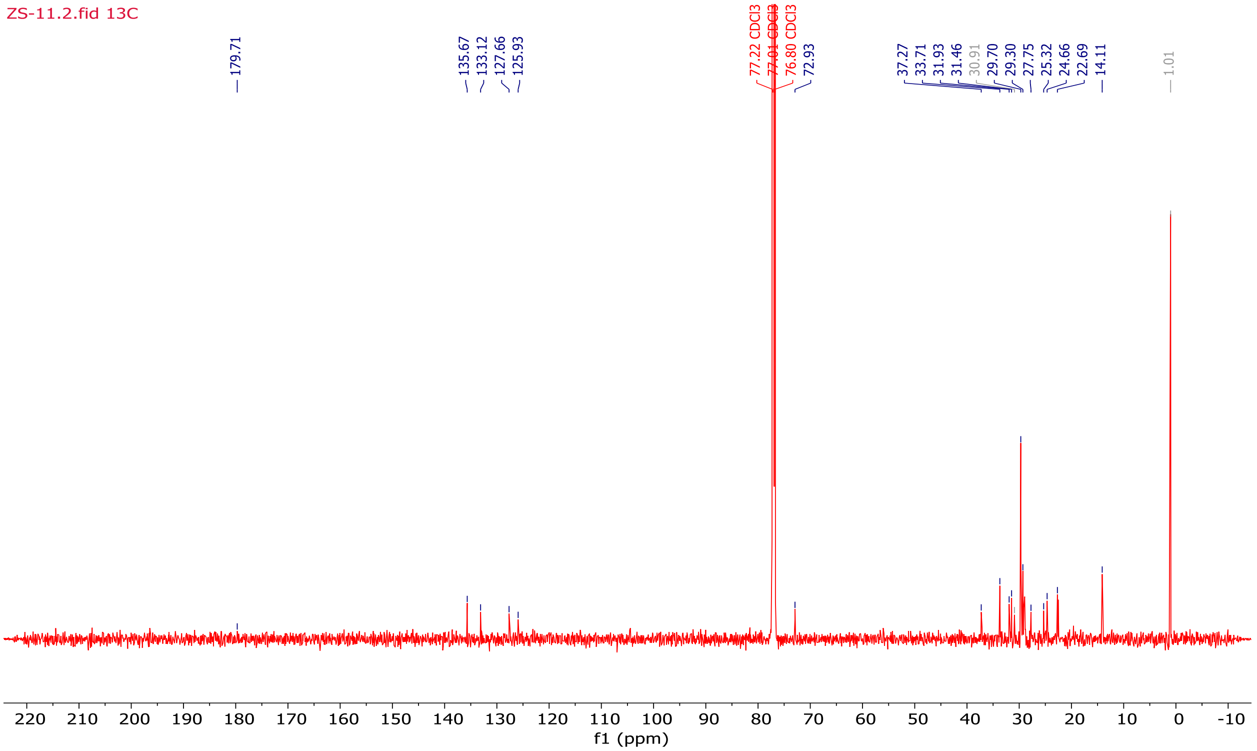

FIGURE S11: ^{13}C NMR spectrum of compound **3**.

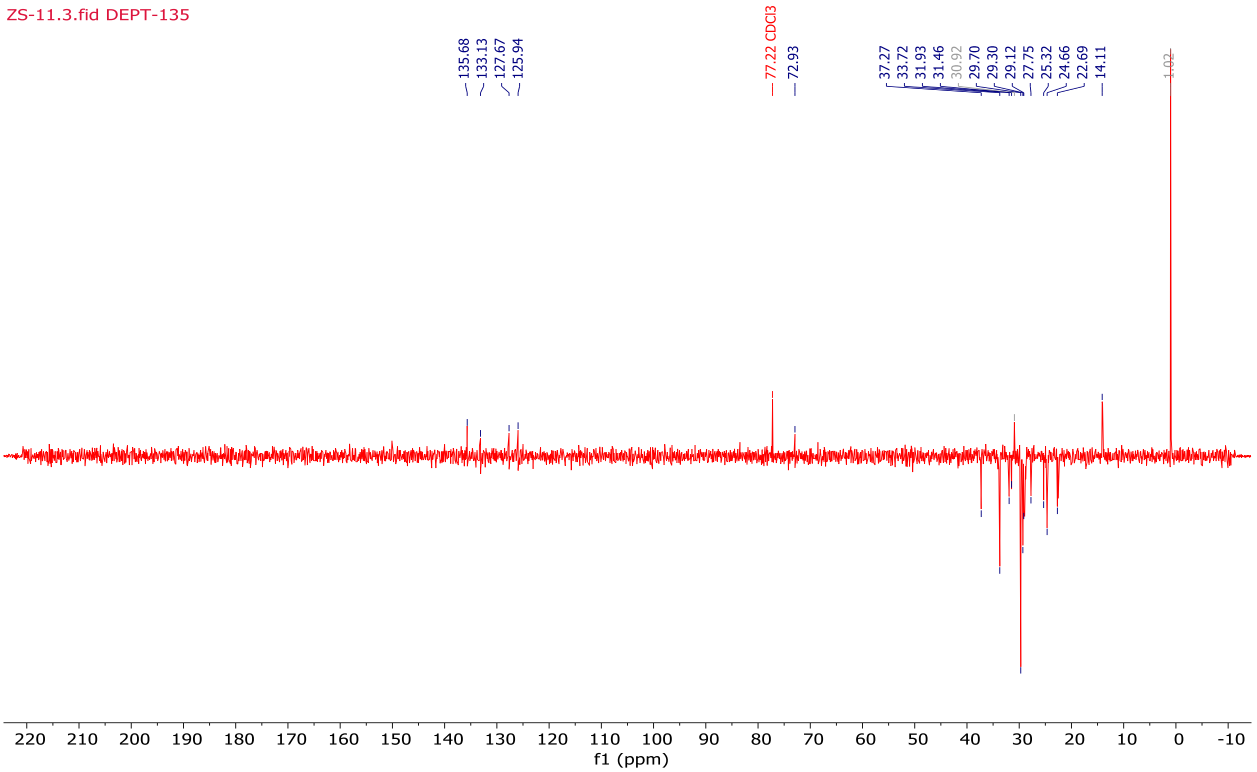

FIGURE S12: DEPT-135 spectrum of compound **3**.

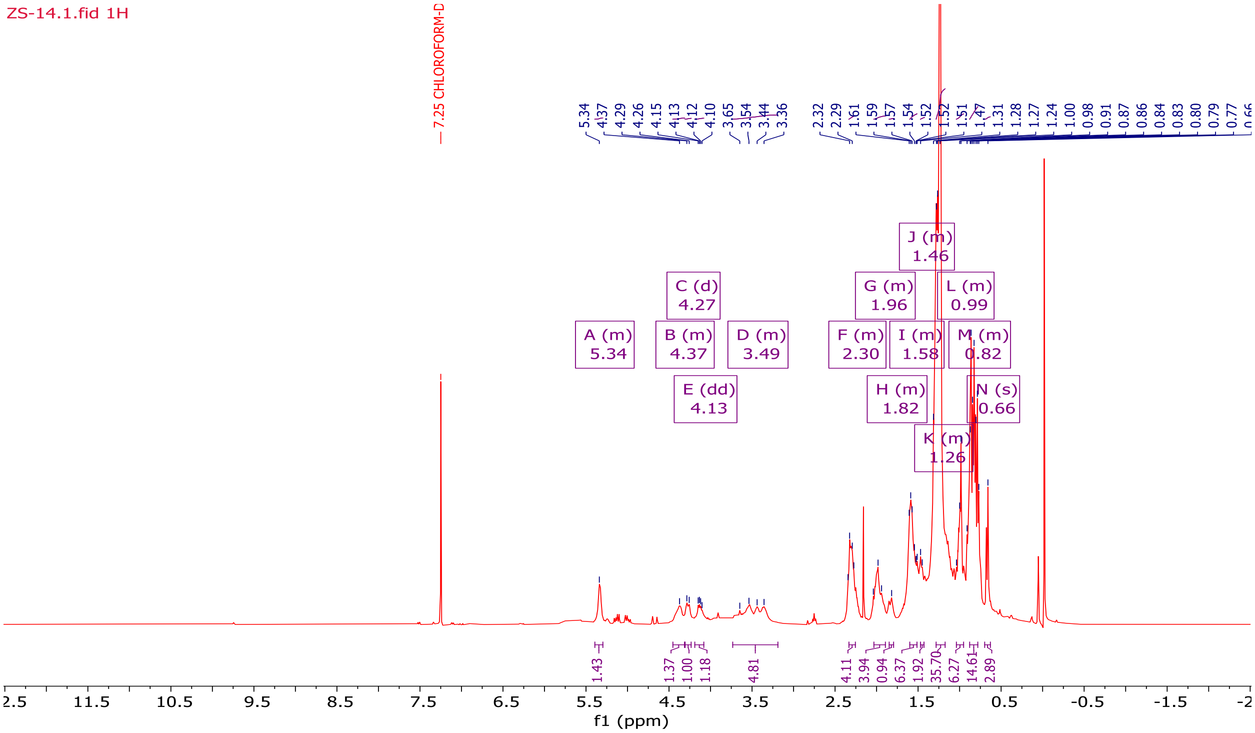

FIGURE S13: ^{1}H NMR spectrum of compound **4**.

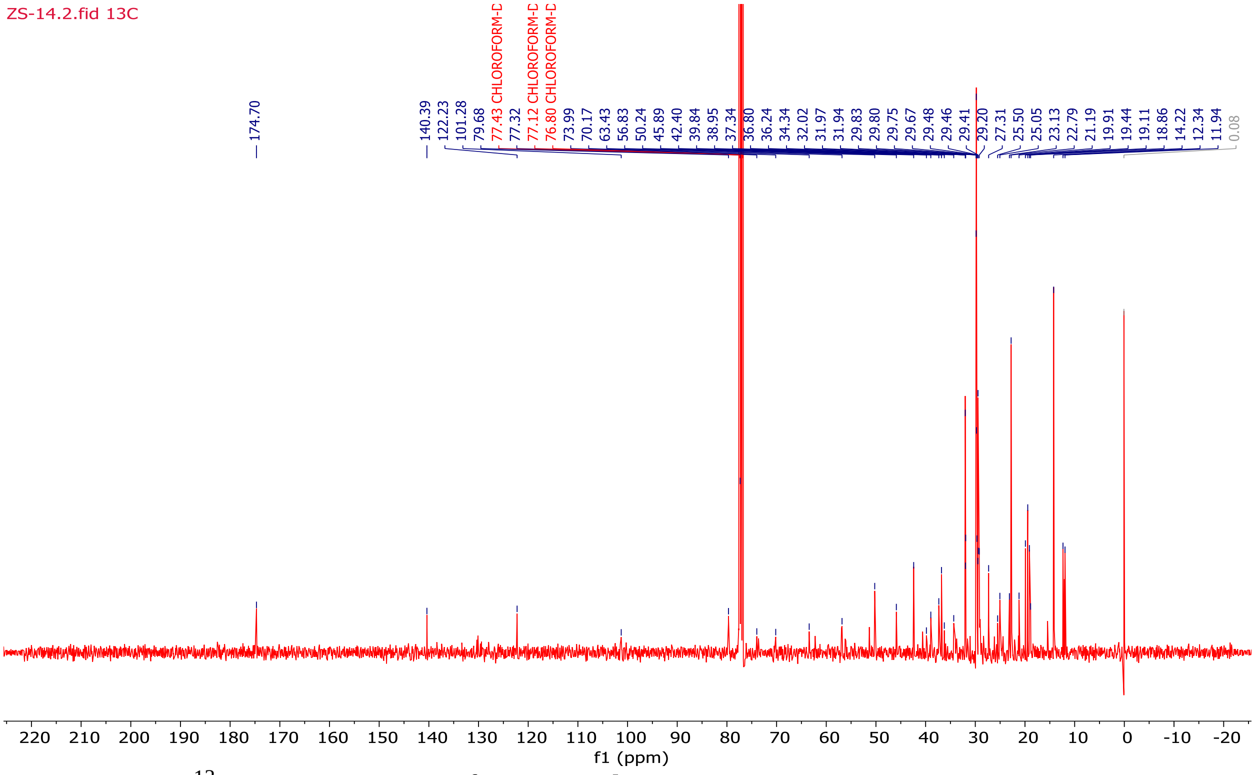

FIGURE S14: ^{13}C NMR spectrum of compound **4**.

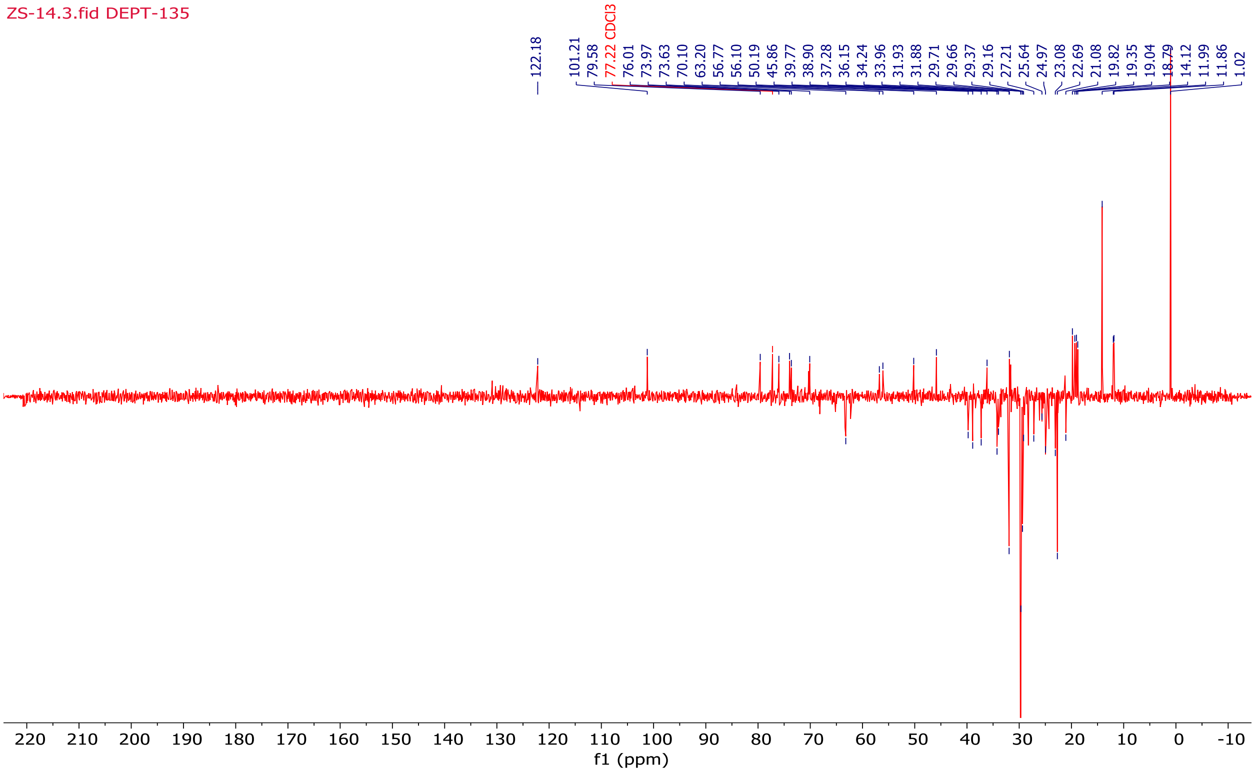

FIGURE S15: DEPT-135 spectrum of compound **4**.

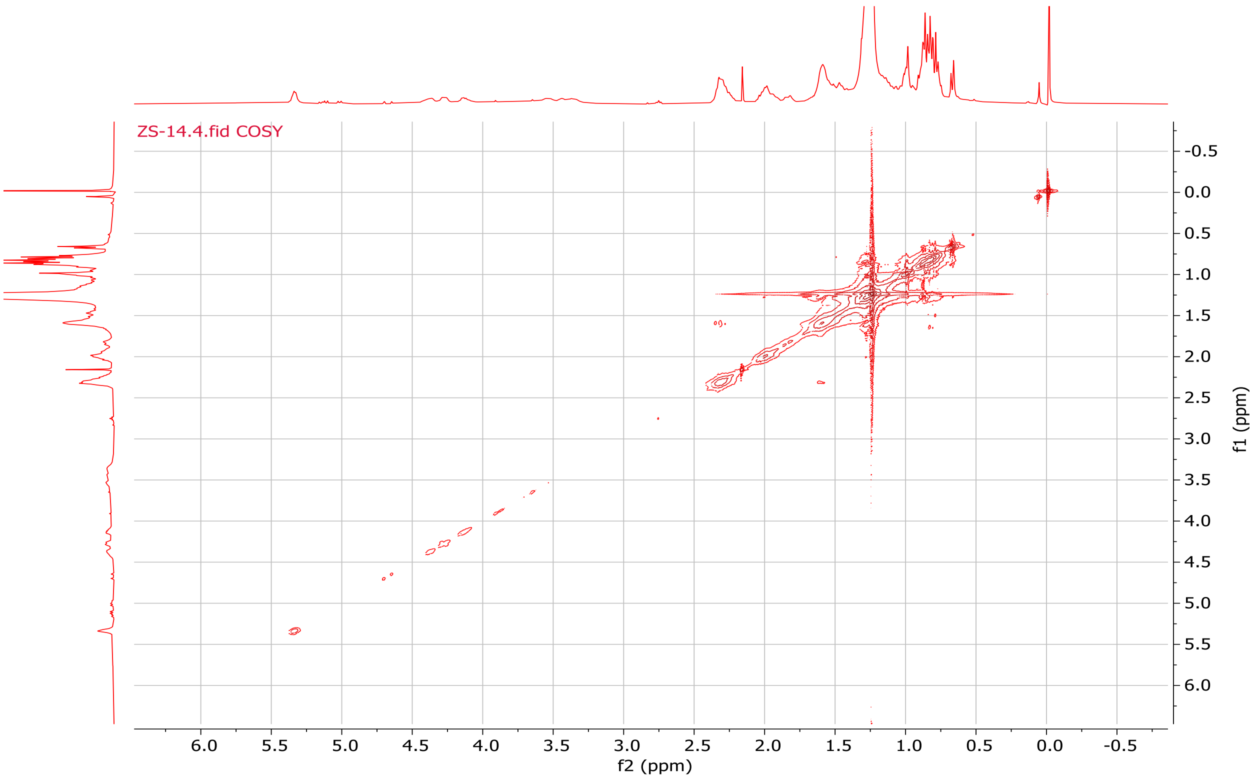

FIGURE S16: COSY spectrum of compound **4**.

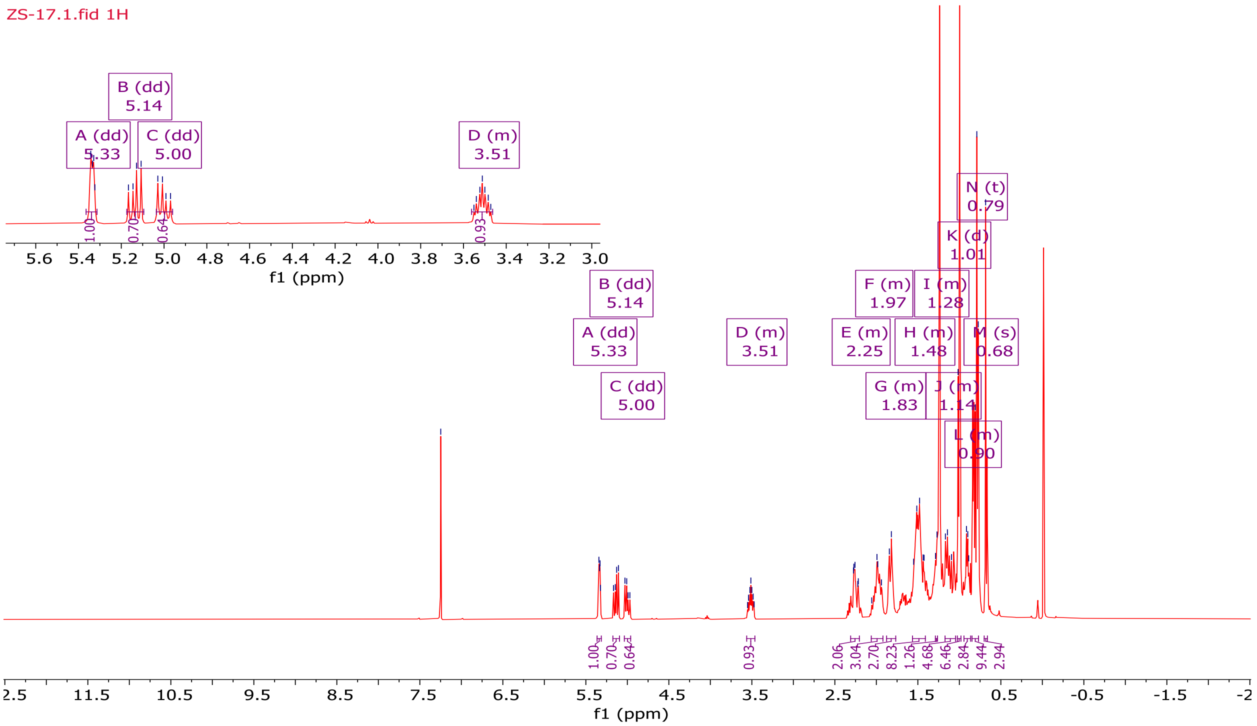

FIGURE S17: ^{1}H NMR spectrum of compound **5**.

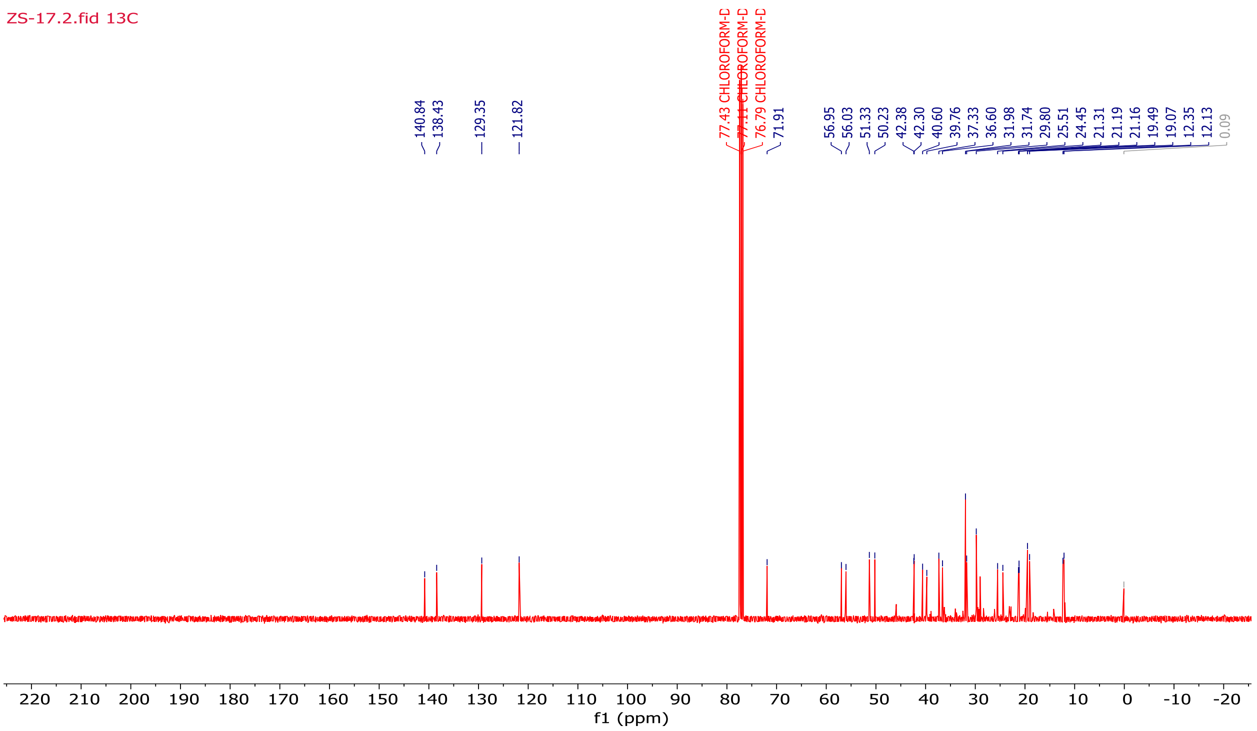

FIGURE S18: ^{13}C NMR spectrum of compound **5**.

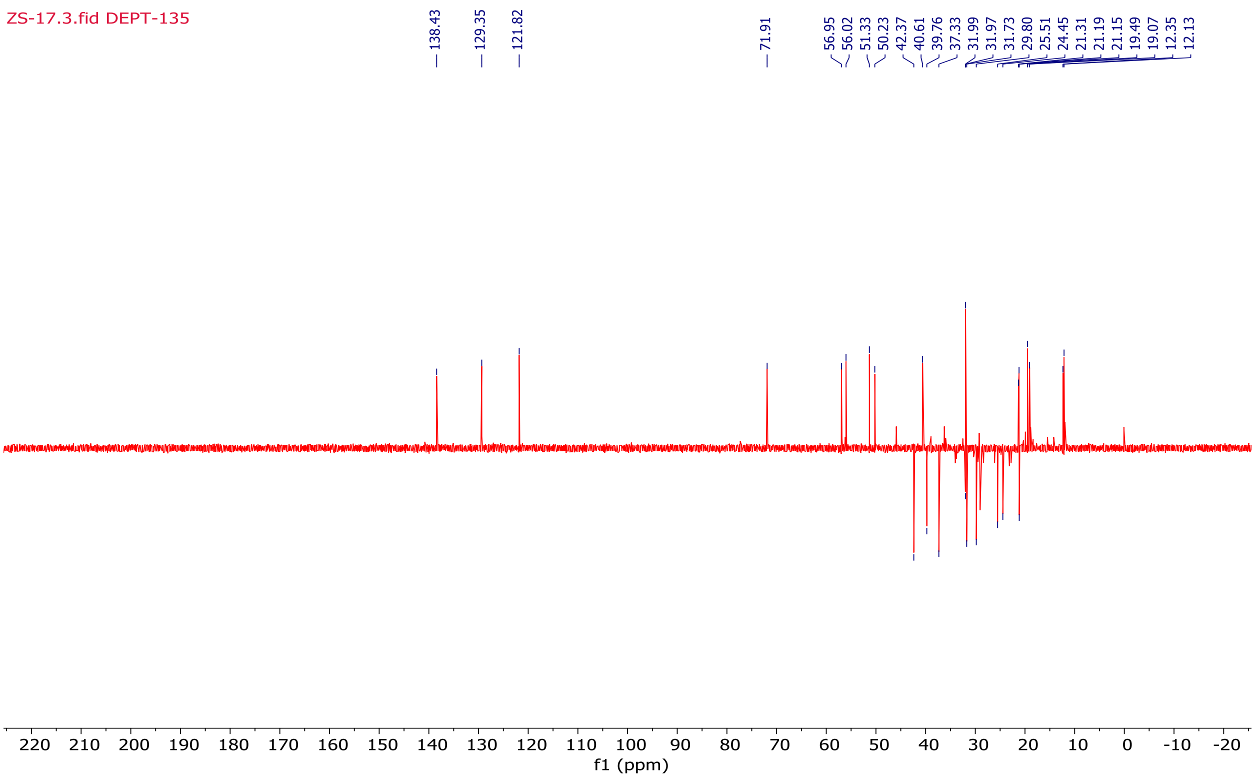

FIGURE S19: DEPT-135 spectrum of compound **5**.

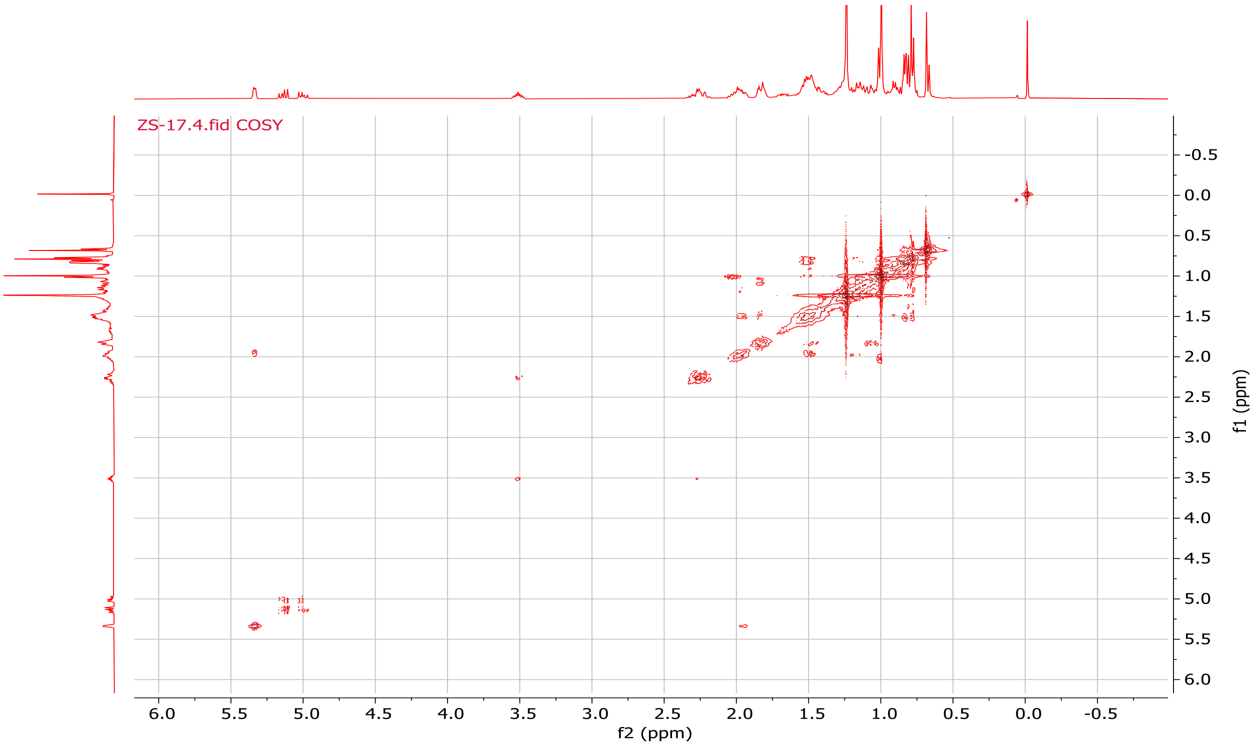

FIGURE S20: COSY spectrum of compound **5**.
